# Effect of Indian Music as an Auditory Stimulus on Physiological Measures of Stress, Anxiety, Cardiovascular and Autonomic Responses in Humans—A Randomized Controlled Trial

**DOI:** 10.3390/ejihpe12100108

**Published:** 2022-10-19

**Authors:** Kirthana Kunikullaya Ubrangala, Radhika Kunnavil, Mamta Sanjeeva Vernekar, Jaisri Goturu, Vijayadas, V. S. Prakash, Nandagudi Srinivasa Murthy

**Affiliations:** 1 Univ Rennes, Inserm, EHESP, Irset (Institut de Recherche en Santé, Environnement et Travail), UMR_S 1085, F-35000 Rennes, France; 2 The Maastricht Multimodal Molecular Imaging Institute, Maastricht University, 6229 ER Maastricht, The Netherlands; 3 National Institute of Unani Medicine, Under Govt. of India, Kottigepalya, Magadi Main Road, Bengaluru 560091, Karnataka, India; 4 Baptist Hospital, Bengaluru 560024, Karnataka, India; 5 Department of Physiology, International Medical School, MSR Nagar, MSRIT Post, Bengaluru 560054, Karnataka, India; 6 Department of Physiology, Ramaiah Medical College, MSR Nagar, MSRIT Post, Bengaluru 560054, Karnataka, India; 7 Department of Cardiology, M S Ramaiah Memorial Hospitals, MSR Nagar, MSRIT Post, Bengaluru 560054, Karnataka, India; 8 Department of Research and Patents, Gokula Education Foundation, MSR Nagar, MSRIT Post, Bengaluru 560054, Karnataka, India

**Keywords:** Indian music, heart rate variability, stress, anxiety, STAI, state anxiety, trait anxiety, blood pressure, melodic modes

## Abstract

Among the different anthropogenic stimuli humans are exposed to, the psychological and cardiovascular effects of auditory stimuli are less understood. This study aims to explore the possible range of change after a single session of auditory stimulation with three different ‘Modes’ of musical stimuli (MS) on anxiety, biomarkers of stress, and cardiovascular parameters among healthy young individuals. In this randomized control trial, 140 healthy young adults, aged 18–30 years, were randomly assigned to three MS groups (Mode/Raga Miyan ki Todi, Malkauns, and Puriya) and one control group (natural sounds). The outcome measurements of the State-Trait Anxiety Inventory, salivary alpha-amylase (sAA), salivary cortisol (sCort), blood pressure, and heart rate variability (HRV) were collected at three time points: before (M1), during (M2), and after the intervention (M3). State anxiety was reduced significantly with raga Puriya (*p* = 0.018), followed by raga Malkauns and raga Miyan Ki Todi. All the groups showed a significant reduction in sAA. Raga Miyan ki Todi and Puriya caused an arousal effect (as evidenced by HRV) during the intervention and significant relaxation after the intervention (both *p* < 0.005). Raga Malkauns and the control group had a sustained rise in parasympathetic activity over 30 min. Future studies should try to use other modes and features to develop a better scientific foundation for the use of Indian music in medicine.

## 1. Introduction

The seven main functions of music are summarized to be background entertainment, recall of memories, diversion, emotion regulation, self-regulation, self-reflection, and social bonding. This umbrella review of the health effects of participation in performing arts, including music participation, reported positive effects in five domains (auditory, cognitive, immune system, self-reported health/wellbeing, and social functioning) [1]. Though music is predominantly used as a form of entertainment the use of music for attaining health benefits dates back over centuries, probably since the Paleolithic period [2]. Music Therapy is defined as the evidence-based use of music as an intervention as per the individual needs of the patient, be it physical, emotional, cognitive, or social, as per the American Music Therapy Association [3]. It is seen that music, when used as an intervention, affects health [4,5] through different processes, which are yet to be well understood. Mechanisms put forth include the impact on the nervous system, the limbic system, [6] the autonomic nervous system [7], as well as synchronization of the body’s natural rhythms (for example, heart rate or respiratory rate) with the rhythm of the music [1,2,8]. Music is a safe, inexpensive, easily administered intervention that can be used for anxiety reduction and has proven to be beneficial in various diseases that include cardiovascular, neurological and oncological diseases, as reviewed in [9,10]. 

The relaxation effects of music on stress, anxiety, and lowering of the neurohumoral markers have been evaluated in several research works [11,12,13,14,15,16,17,18]. Active music intervention has proven to be beneficial in people afflicted with post-traumatic stress disorder [19,20]. Young people report that music can help them relax and often have a collection of favorite ‘tunes’ that they listen to when they feel stressed out [15,21]. In a cross-sectional study, we observed a significant drop in state anxiety after listening to Indian music [22], and in another follow-up study, we observed a significant reduction in state and trait anxiety after 3 months of Indian music intervention on 100 pre-hypertensives and hypertensives [23]. Recent meta-analysis reports show that music is efficient in reducing anxiety levels [24,25], though some emphasized the need for additional research to endorse the same [26]. Among the physiological measurements that can be correlated with anxiety and stress are the cardiovascular parameters such as blood pressure (BP) and autonomic functions (that control the heart). Music-based interventions have largely been carried out on patients with hypertension [23,27,28,29,30,31] studying cardiovascular effects or perioperative conditions [25,32,33,34,35] studying anxiety. Listening to sedative music decreased heart rate (HR) and BP and was shown to work as effectively as benzodiazepines in reducing BP [36].

Heart rate variability (HRV) is a marker of cardiac autonomic functions [37] and has also been commonly investigated in music intervention studies [38]. Several studies reported decreased HRV, indicating physiological relaxation [33,39,40,41,42], while some reported no change [43], and a few others showed an increase in HRV on listening to music (arousal effect) [40,44]. The overall effect also depends on the mood [21], preferences [45], tempo, genre, and various other factors [40], for example, listening to preferred music caused increase in sympathetic activity, regardless of the type of music (calming or stimulating) [46]. It is important to understand the genre and features of the music used in a study before interpreting the HRV findings. It may also be observed that there is evidence of music’s varied effects on stress, anxiety, the cardiovascular system (BP and HRV), and the mechanisms behind it. Although a subjective reduction in stress levels was recorded, objective measurements [47,48] or psychophysiological signals have not always shown the same [49,50]. A systematic review and meta-analysis also failed to establish a cause-effect relationship between the intervention and BP reduction [51]. It is thus important to have both subjective and objective measurements during music intervention to draw reasonable conclusions.

Among the music features, works on the effect of listening to different modes/melodic scales to elicit the difference in physiological effects, if any, are very few. Among the different genres, very little literature is available on Indian music as a scientific intervention, despite the rich repertoire of modes in this genre, for producing relaxation effects or health benefits [22,52,53,54,55,56]. Indian music is broadly classified into Hindustani and Carnatic music, each having its system of modes (called *ragas*). Ancient literature on Indian music (*Gandharva Veda*, a part of the *Sama Veda* and ‘*Raga Chikitsa*’ manuscript) mentions various modes that have health benefits [23,57,58]. A ‘*raga*’ (melodic mode) is a set of musical notes presented in an orderly manner to generate a melody out of the same and has the “effect of coloring the hearts of men” [22,52,58,59]. Each melodic mode is said to induce a specific emotion (called ‘*rasa*’) [58,60,61]. Scientific studies that have analyzed emotions after listening to Indian classical music have observed that the tonality of the scale is an important factor that determines the emotions perceived [52,54,55]. To the best of our knowledge, not many studies have included behavioral parameters with physiological measurements while listening to different modes of Indian music.

With this purview, this study tried to elucidate the effects of listening to Indian classical music on different behavioral and physiological parameters among young healthy individuals. Music is a complex stimulus that unfolds over time, it is important to understand the effect of systematically combined musical features during an average duration of listening. Our specific hypothesis was that distinct cardiovascular and behavioral responses would be associated with passive listening to each specific auditory stimulus, the response being specific to the melodic mode. For this, we chose three Indian modes (*ragas—Puriya, Malkauns*, and *Miyan ki Todi*). The primary outcome measure was to evaluate the state and trait anxiety levels, biomarkers of stress, blood pressure, and autonomic functions (HRV) after short-term listening to pre-recorded music in each of the three modes mentioned above.

## 2. Materials and Methods

### 2.1. Study Design

A prospective, parallel-group, triple-blinded, randomized controlled trial was conducted with an experimental study design, with a sample of 140, randomized into 4 groups, A to D, with a sample of 35 participants in each group. The four acoustic stimuli (stored as .mp3 files) were coded by a person uninvolved in the current study as A, B, C, and D, to be used as respective group interventions.

### 2.2. Ethical Approvals

The study protocol was approved by the institutional scientific committee on human research and ethical review board (Reference: MSRMC/EC/2017, dated: 25 July 2017). The study period ranged from 2019 to 2021 (June 2019—first recruitment and February 2021—last recruitment). The data presented here were taken from a larger experiment (full trial protocol: NCT03790462 on clinicaltrials.gov.in). The research was conducted following the Declaration of Helsinki guidelines [62].

### 2.3. The Basis for Sample Size

After music intervention, the State-Trait Anxiety Inventory-6 (STAI-6) anxiety scores changed from 33.3 (23.3–41.7) to 30 (20–40), respectively (Median [interquartile range—IQR]), in a previous study [63]. Using these data, considering the minimum difference of 4 units in the STAI score before and after the intervention, with an effect size of 0.7, power of 85%, and an alpha error of 5%, the sample size was calculated to be 35 in each group.

### 2.4. Recruitment

The study participants were recruited from a group of educational institutions in the city of Bengaluru, Karnataka, India. Healthy Indian individuals aged 18–30 years were invited to participate in the study via an open call for participants for the study posted online (social media) and notice board advertisements across the institutions. Given the objectives of the study, to avoid cultural familiarity differences, only Indians were invited to participate in this study. Participants who responded to the call were sent an online questionnaire via Google forms. 

### 2.5. Inclusion and Exclusion Criteria

Inclusion criteria were participants volunteering for the study, aged 18–30 years, of either gender and medically and surgically healthy individuals (initially self-reported—based on the online questionnaire and later confirmed on visiting the lab). The participants had to be non-smokers and non-alcoholics. Participants on any medication (based on drug intake history, drugs known to affect the BP or autonomic status of the individual) were excluded from the study. Pregnancy and body mass index (BMI) > 30 kg/m^2^ were the other exclusion criteria.

### 2.6. Baseline Demographic Data Recording

A web-based questionnaire (Google forms) was designed and implemented for this study. This questionnaire contained details such as a unique identification number for each subject, subject’s name, gender, socio-demographic details, education background, drug history, present report or history of non-communicable diseases if any, and family history of non-communicable disorders, and smoking and alcohol history. A questionnaire containing details inquiring about the participants’ preference for any type of music and previous experience with music (instrumental or vocal training) was also included. After screening >300 individuals who responded to the call, 166 completed the Google form. Following the collection of data online, the participants were invited for further data collection in the lab. Out of 166 participants who answered the online questionnaire, 154 participants reported to the lab. The principal investigator (PI) and Co-PI enrolled participants in the study.

### 2.7. Randomization

**Figure 1:**
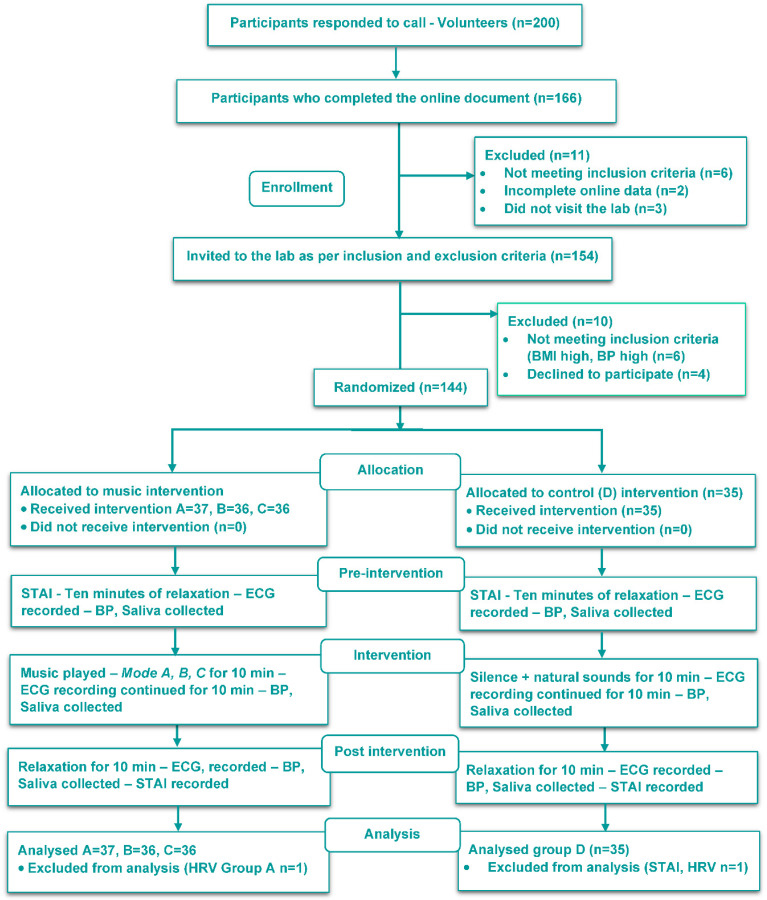
Consort diagram of participant recruitment, distribution, and follow-up.

The total sample size (*n* = 140) was randomized into 4 groups using a simple randomization technique where the random numbers were computer-generated using MS Excel (4 sets of 35 each). The numbers generated were kept in a sealed, opaque envelope which was opened by the research assistant after the baseline assessment of each participant who assigned them to each of the four groups (Consort diagram Figure 1).

### 2.8. Interventions

Three groups (A, B, C) received one of the *ragas*/modes as an acoustic intervention, while the fourth group (Group D/control arm) received natural sounds as an acoustic stimulus (all audio clips were coded as A, B, C, and D by a person uninvolved in the study).

#### 2.8.1. Music Intervention

Ten-minute, tailor-made instrumental renditions of 3 modes were digitally pre-recorded and played via headphones [64] connected to a laptop, at a uniform volume (50%). Group A received *raga Miyan ki Todi*, group B received *raga Malkauns*, and group C received *raga Puriya* [Table 1]. These modes were chosen based on the criterium of having beneficial cardiovascular effects as per ancient music literature [23,57,61,65]. The music was tuned to be at a frequency of 329.63 Hz (the tonic or ‘*Sa*’ at Pitch E). Details about each mode used and the notes can be found in the Supplementary File (S). The start and end time of the music was marked using an event marker in the software. 

We instructed the participants to listen to this with eyes closed, and minds relaxed, for the duration it was played. The music was recorded by an eminent musician in India (exclusively for the present study) with the drone (*tanpura*) in tonic in the background and flute/*Bansuri* playing the respective *alaap* in the above-mentioned scales. A specific rhythmic structure or tempo was not there for this musical piece, and percussion instruments were avoided. The ‘*Bansuri*’ is a flute in India made from a single hollow shaft of bamboo with six or seven finger holes, held horizontally while playing [66]. As there was very little literature available on the most relaxing or soothing instrument, we chose *bansuri* for this study based on common instruments used commercially to produce relaxing music tapes. Instrumental music helped us avoid percussion (tempo) [67,68,69,70,71,72,73,74], lyrics, and the emotions or semantic processing due to them, and thus the music had minimal pitch dynamics, contrasts, and rhythm in it.

#### 2.8.2. Control Group Intervention

The control group (Group D) did not receive any music intervention, but since the complete recording lasted for 30 min duration, it was possible for the participants to feel sleepy (sleep is anxiolytic, which would alter the current objective). Thus, natural sounds (birds chirping and flowing river) were played for 10 s duration once every 2 min in the middle ten min (during intervention); a total of 50 s in the middle ten minutes. This also ensured uniformity of intervention between the groups.

### 2.9. First Visit to the Lab

Before visiting the lab, all participants were instructed to come after overnight fasting, with a light breakfast, to abstain from tea and coffee about 2 h before the recording, and abstain from exhaustive exercise, for the preceding 24 h. Female participants were asked to visit the lab during the follicular phase of their menstrual cycle. The study protocol and the rights to withdraw their participation from the study were explained to the participants, after which written informed consent to participate in the study was obtained. A general health check-up was done for all participants. The BMI was calculated, and BP was measured twice. The healthy cardiovascular system of the volunteers was defined by measuring BP, which confirmed their non-hypertensive state, and by measuring baseline HR, which confirmed their non-tachycardiac state. Normotensives were included as per inclusion criteria (excluded *n* = 10; Baseline systolic BP—SBP > 120 mm Hg). Healthy participants (*n* = 144) were recruited (Consort diagram Figure 1). Though 10.4% of participants were current alcoholics, their baseline BP was within the normal range and were included in the study after being instructed to abstain from smoking/alcohol 24 h before the recording.

### 2.10. Second Visit to the Lab

**Figure 2:**
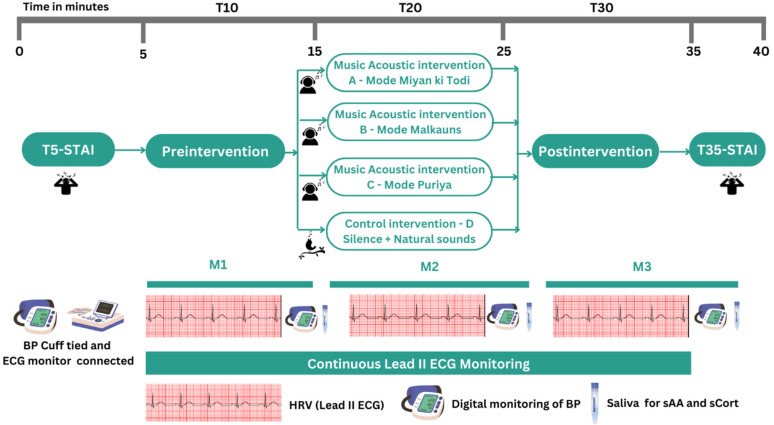
Study protocol; T5, T10, T20, T30, T35 is the time in minutes; STAI—State-Trait Anxiety Inventory; BP—Blood pressure; HRV—Heart rate variability; ECG—Electrocardiogram; sAA—Salivary Alpha-amylase; sCort—Salivary Cortisol.

All the recordings were carried out between 09:00 and 11:00 a.m. in an isolated room at a stable temperature between 20 and 22 °C, in a noise-free atmosphere. After the participants responded to the STAI Form Y (between 0 to 5 min—T5, explained further), they were asked to relax in the supine position for 10 min before the tests, with their eyes closed. Participants were carefully monitored to ensure there were no significant respiratory or postural changes during the session. During this time (first 5 min), electrocardiogram (ECG) electrodes in lead II were applied, similar to previous studies [64], headphones were adjusted, and comfort with the pre-set volume (50% on laptop) was tested. The BP cuff was tied to the left arm of the participant and a reading was taken so that the participant understands the process of automatic cuff inflation and deflation. At the end of the first 10 min the baseline BP was recorded, and the protocol was begun. At the beginning of the protocol, one saliva oral swab was inserted into the participants’ mouths, kept sublingually. The first 10 min of baseline ECG recording commenced. At the end of 10 min (M1), digital measurement of BP (SBP, diastolic BP—DBP, and HR) was done (recorded as pre-intervention readings) and saliva samples were taken. This was repeated at 20 (M2) and 30 (M3) minutes later (see Figure 2 for the process of recording). After the 30 min protocol, the participants were asked to complete the STAI Form-Y (between 35–40 min—T35), recorded as post-intervention STAI scores, and rate the valence of intervention on a 10-point visual analog scale (VAS). All saliva swabs were stored at 4 °C until centrifugation. The saliva samples were then centrifuged at 3000 rpm for 15 min and supernatant saliva was stored at −80 °C until further analysis within 1 h of saliva collection. Pre, during, and post-intervention data analysis of BP, HRV, STAI, and salivary stress markers (salivary cortisol—sCort and salivary alpha-amylase—sAA (ELISA)) was done.

### 2.11. Behavioral Measures

#### Measurement of Anxiety

The State-Trait Anxiety Inventory (STAI) (Form Y) for adults is a validated questionnaire [75] implemented in the current study, as explained in [22]. Briefly, participants had to respond to 40 questions by rating themselves on a four-point Likert scale (1—Not at all, 2—Somewhat, 3—Moderately so, 4—Very much so), resulting in a range of possible scores between 20 to 80 on both the State and Trait subscales [75]. It differentiates between the temporary condition of “State anxiety” (feeling at the moment, in Form Y 1) and the more general and long-standing quality of “Trait anxiety” (feeling in general, in Form Y 2) [75,76]. The STAI has demonstrated good internal consistency (average as >0.89) and test–retest reliability (average r ¼ 0.88) at multiple time intervals. The reliability of the STAI in patients with an anxiety disorder is found to be between 0.87 and 0.93 [77,78]. One of the STAI forms in group D had more than 3 missing values and was thus not included for further analysis.

### 2.12. Physiological Parameters

#### Saliva for Biomarkers of Stress

To measure free cortisol levels, which reflects hypothalamic–pituitary–adrenal system (HPA) activity, and salivary alpha-amylase (sAA), which indicates the activity of the sympathetic–adrenal–medullary system (SAM) [79,80,81], saliva was collected using the SalivaBio Oral Swab (Salimetrics LLC, State College, PA, USA) every 10 min (Figure 2). Salivary alpha-amylase was assessed using a Salimetrics Salivary Alpha-Amylase Assay Kit (Salimetrics LLC, State College, PA, USA), following the manufacturer’s guidelines. Results were expressed in U/mL. The intra-assay precision coefficient of variation (%) was 2.5–7.2%, and the inter-assay precision was 3.6–5.8%. Salivary levels of cortisol were assessed using the Expanded Range High Sensitivity Salivary Cortisol Enzyme Immunoassay Kit (Salimetrics LLC, State College, PA, USA), following the manufacturer’s guidelines. Results were expressed in µg/dL. The intra-assay coefficient of variation was 5.5–5.68%, and the inter-assay coefficient of variation was 6.3–6.7%.

### 2.13. Cardiovascular Parameters

#### 2.13.1. Blood Pressure (BP) and Heart Rate (HR)

A standardized digital BP monitor was used (Omron HEM-7130-L, OMRON Healthcare Manufacturing Vietnam Co., Ltd., Sourced from Haryana, India) to measure BP [82]. The measurements of SBP (in mm of Hg), DBP (mm Hg), and HR in beats per minute were noted once every 10 min (Figure 2).

#### 2.13.2. Electrocardiogram Recording and Heart Rate Variability Analysis

The Electrocardiogram (ECG) was recorded in Lead II (sample rate of 1000 Hz) for ten minutes as this is twice the minimum window required for HRV analysis. The data were recorded using Power lab 15 T LabChart Pro 8 software (ADInstruments, Sydney, Australia) and analyzed as described in [37]. Analysis of HRV was done by the same investigator to avoid sources of error. The HRV parameters analyzed using fast Fourier transformation (FFT size: 1024) were SDNN—the standard deviation of NN intervals, RMSSD—root square of the mean squared difference of successive NNs, NN50—number of pairs of successive NNs that differ by more than 50 ms, pNN50—the proportion of NN50 divided by the total number of NNs, spectral components such as Very Low-Frequency (VLF), Low-Frequency (LF), and High-Frequency (HF) components in absolute values of power (ms^2^) and normalized units (nu), and LF/HF. Pre (M1), during (M2), and post-intervention (M2) parameters of HRV (as an average of a minimum of 5 min of recording, during each condition) were analyzed. During analysis, one of the HRV readings was not saved in group A and one in group D was too noisy, and they were thus not analyzed and were deleted from further processing.

### 2.14. Statistical Analysis

Analysis was conducted at three levels: (1) group-wise (within and between) behavioral analysis of anxiety scores and stress markers, (2) group-wise (within and between) analysis of cardiovascular responses, and (3) regression analysis to investigate the relationship between the acoustic stimulus used and the cardiovascular and behavioral responses. Data were analyzed using SPSS software version 18.0 software (SPSS Inc. Released 2009. IBM SPSS Inc., Chicago, IL, USA). The continuous variables were analyzed using descriptive statistics such as mean and SD or median and interquartile range as per skewness of data. The qualitative/categorical variables were analyzed using frequency and percentage. The normality of the BP and HRV data was checked by applying the Kolmogorov–Smirnov Test. The categorical variables were tested for differences in proportion using the Chi-Square test of significance. Pre- and post-intervention data analysis of state and trait anxiety scores were compared using Wilcoxon’s signed-rank test. The Independent *t*-test was used to compare the differences between the groups. Baseline comparisons were carried out using a one-way analysis of variance (ANOVA). BP, HRV, and salivary parameters were compared across different groups pre, during, and post-intervention using repeated measures of ANOVA (RM-ANOVA). The HRV parameter’s absolute levels and log-transformed levels were compared using RM-ANOVA with sphericity assumption. Further, a two-way RM-ANOVA analysis was done to inspect the interaction between the intervention group and time. Analysis of covariance (ANCOVA) was used to assess the effect of various covariates, viz., age, age groups, gender, smoking, alcoholism, involvement in mind-body relaxation techniques, physical activity, and music training, on the change in STAI, BP, and HRV parameters over time. Apart from tabulation, data were also depicted graphically using box plots and line diagrams. A two-tailed *p*-value < 0.05 was considered statistically significant at a 5% level of significance.

## 3. Results

### 3.1. Sociodemographic Data

The sociodemographic data showed that the groups were comparable (Table 2), except for their educational status. There were more graduate students in the music intervention groups compared to the control groups (*p* < 0.001). About 30 to 45% of participants were trained in music, but the distribution of participants across the groups was comparable. Participants were predominantly trained in Indian music, with more than 70% trained for more than a year. About 85% of participants considered themselves familiar with or experts in Indian classical music (Supplementary File Table S1).

### 3.2. Behavioral Analysis

#### STAI

**Figure 3:**
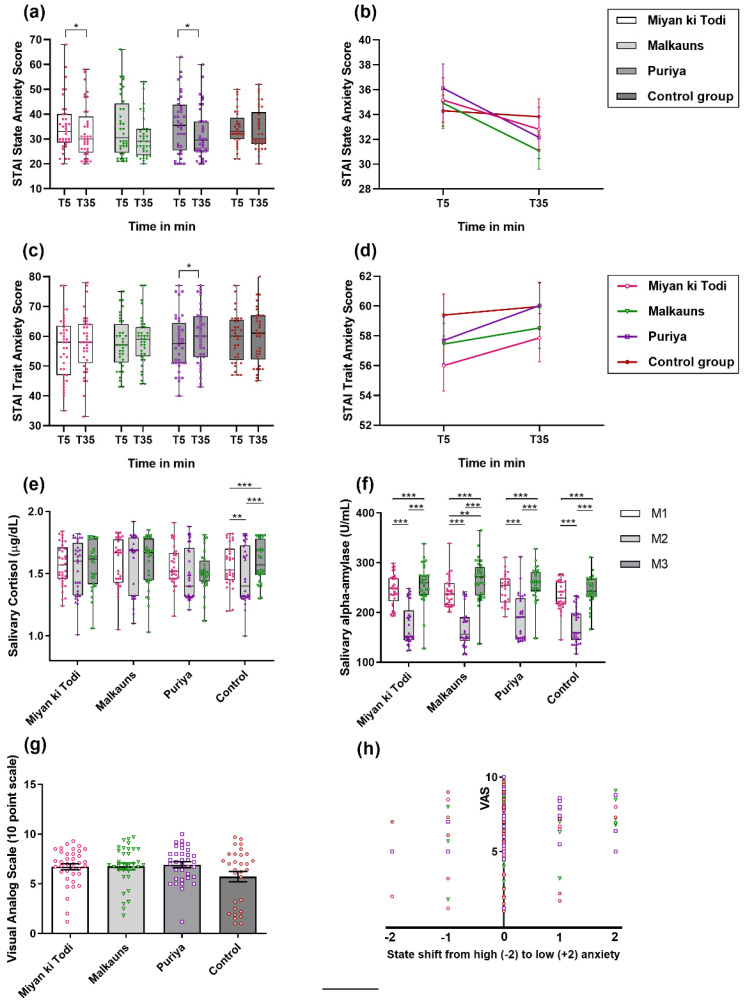
(**a**,**b**) Comparison of state score on STAI-Y1; (**c**,**d**) Trait score on STAI-Y1; (**e**) Salivary stress markers—Cortisol in μg/dL, (**f**) Alpha-amylase in U/mL, (**g**) VAS (valence rating), and (**h**) change in state anxiety score with VAS scoring—among the four groups at different time points (T5 is 5 min before the protocol began, and T35 is 35 min after the protocol or 5 min after the protocol was completed). Note: *: *p* < 0.05, **: *p* < 0.01, *** *p* < 0.001.

Pre-intervention levels of the state and trait score (T5), across the groups, were comparable (for state, *F*(3, 138) = 0.170, *p* = 0.917; and for trait, *F*(3, 138) = 0.811, *p* = 0.490). Comparison of state anxiety STAI scores between all three music intervention groups showed statistical significance (reduction) within the group. The maximum reduction in state score was with *raga Puriya* (mead difference—md = 3.94, *p* = 0.018), the next being *raga Malkauns* (md = 3.83, *p* = 0.057), followed by *raga Miyan ki Todi* (md = 2.35, *p* = 0.054). The reduction in the control group was mild, md being 0.32 (statistically not significant) (Table 3, Figure 3a,b). Between the groups, there was no significant difference in the T35 state score (*p* = 0.696). On comparison of the difference in means of pre–post values between the groups, there was no significant difference in state score (*p* = 0.319). A comparison of trait anxiety scores showed that group C (*raga Puriya*) had a statistically significant increase in trait score (increase by 2.33 mean level, *p* = 0.011) (Table 3, Figure 3c,d). Between the groups, the T35 trait score was not significant (*p* = 0.660). There was no significant difference in the pre–post values of trait scores between the groups (*p* = 0.634). On multivariate analysis, none of the confounding variables seemed to affect the change in STAI scores (both state and trait).

### 3.3. Physiological Parameters

#### Biomarkers of Stress

Pre-intervention levels of sAA and sCort (M1), across the groups, were comparable (for sAA, P, *F*(3, 127) = 1.421, *p* = 0.240; for sCort, *F*(3, 126) = 0.197, *p* = 0.898). Mean sCort levels reduced maximally in the control group (F = 12.34, *p* < 0.0001). Mean sAA levels reduced in all four groups significantly at M2, after which the levels increased slightly more than baseline levels. The drop in sAA was maximal at M2 with *raga Puriya* (F = 67.01, *p* < 0.0001), which increased at M3 to a level higher than within the group baseline and in comparison with other groups (Figure 3e,f; post hoc analysis in the Supplementary File, Table S2). The visual analog score and corresponding state scores did not vary significantly across groups (Figure 3g,h).

### 3.4. Cardiovascular Parameters

#### 3.4.1. Blood Pressure and Heart Rate

**Figure 4:**
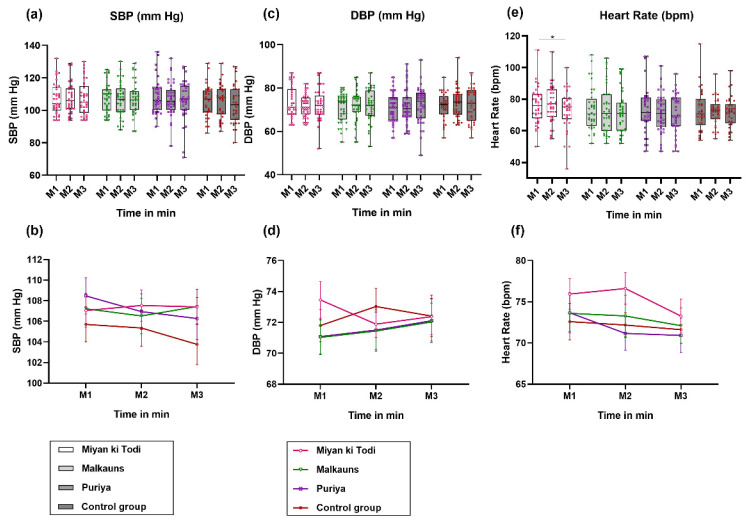
Comparison of (**a**,**b**) systolic BP (SBP), (**c**,**d**) Diastolic BP (DBP), and (**e**,**f**) Heart rate among the four groups at different time points (M1 is at the 10th minute, M2 is at the 20th minute, M3 is at the 30th minute). Note: *: *p* < 0.05.

Pre-intervention levels of BP and HR (M1 levels), across the groups, were comparable (for SBP, *F*(3, 139) = 0.463, *p* = 0.708; for DBP, *F*(3, 139) = 1.053, *p* = 0.371; for HR, *F*(3, 139) = 0.417, *p* = 0.741). On RM-ANOVA analysis of the intervention effects, no significant differences were observed in SBP and DBP in any of the groups (Figure 4, explanation elaborated in Supplementary Text S4). Heart rate increased with *raga Miyan ki Todi* intervention and reduced below baseline levels at M3 (F = 3.645, *p* = 0.031), with maximum difference seen between M2 and M3 (mean difference = 3.351 drops, *p* = 0.073) (detailed in Supplementary File, Table S3a,b). It may be observed that, in line with the STAI state anxiety, SBP and HR reduced maximally with *Raga Puriya*.

On multivariate analysis, it was found that age and physical activity had a significant effect on change in the mean SBP. There was a statistically significant effect of age group on SBP, *F* (1, 132) = 5.572, *p* = 0.020, and involvement in physical activity on SBP, *F* (1, 132) = 4.664, *p* = 0.033. However, the percentage of variation in SBP that could be explained by the independent variables mentioned in the table was only 10% (R Squared = 0.107). On gender-wise and subgroup analysis based on involvement in physical activity, no significant differences were observed in BP or HR levels. The particular age group of 22–24 years old showed a significant effect (F = 3.308, *p* = 0.043), based on time (i.e., M1 vs. M2 vs. M3 SBP levels); however, group-wise means difference was statistically not significant. On comparison of DBP levels based on age groups, we observed that participants aged 18 years showed significant changes in DBP based on time (F = 7.337, *p* = 0.002) and interaction effect (time and group, F = 2.773, *p* = 0.024). A subgroup analysis based on training in music failed to show significant differences in BP or HR.

#### 3.4.2. Heart Rate Variability

All pre-intervention HRV parameters across the groups were comparable (all HRV parameters had *p* > 0.05, data in Tables S4 and S5, Supplementary File), except for VLF ms^2^ (high in group C and low in group A, *F* (3, 138) = 2.878, *p* = 0.038). The comparison of intervention was done using RM-ANOVA (for actual values and statistics, see the Supplementary File, Tables S4–S6).

##### Time-Domain Parameters of Heart Rate Variability

**Figure 5:**
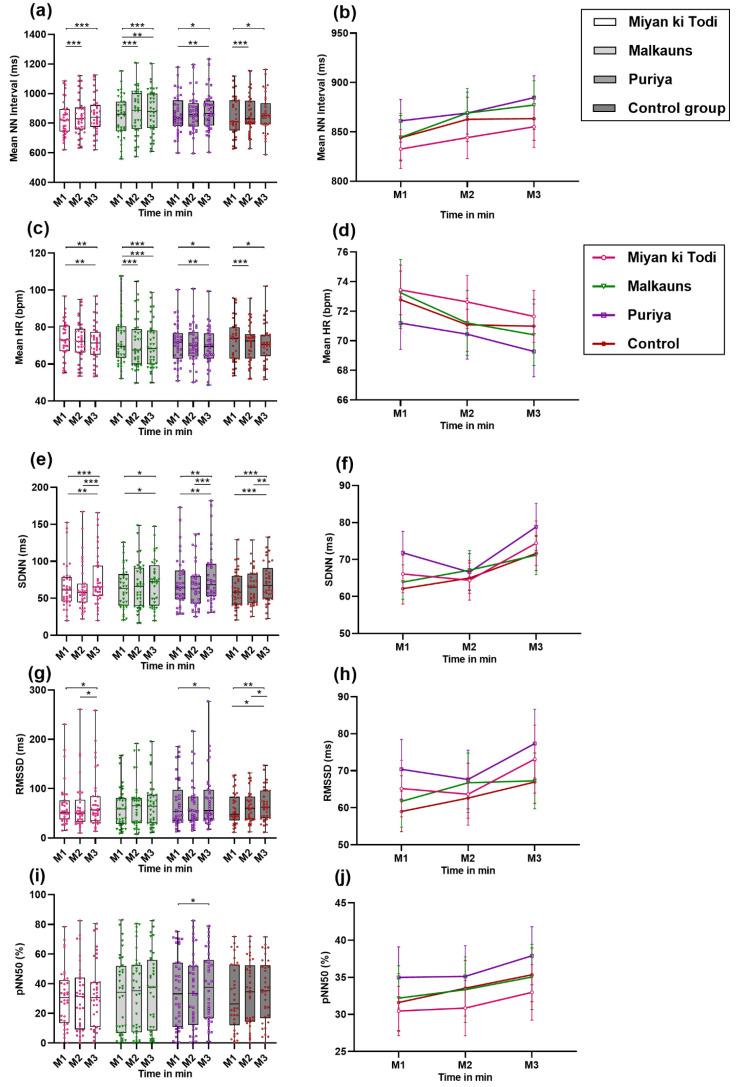
Comparison of Time domain parameters of HRV among the four groups at different time points ((M1, M2, M3 is the measurement of HRV pre-intervention (T10), during the intervention (T20), and post-intervention (T30)). (**a**,**b**) Mean NN interval in ms; (**c**,**d**) Mean HR (bpm); (**e**,**f**) SDNN in ms; (**g**,**h**) RMSSD; (**i**,**j**) Percentage of NN50 in %. Note: * *p* < 0.05, ** *p* < 0.01, *** *p* < 0.001.

There was a continuous rise in mean NN among the music intervention groups through the 30-min protocol, but in the control group, the change was minimal at M3 (last 10 min, after intervention). Post hoc comparisons revealed a significant rise from M1 (first ten minutes before intervention) to M3 mean NN with *raga Miyan ki Todi* (difference of 22.67 ms; *p* < 0.001), *raga Malkauns* (difference of 33.15; *p* < 0.001), and *raga Puriya* (difference of 23.46; *p* = 0.01). Group listening to *Raga Malkauns* (difference of 24.78; *p* < 0.001) and the control group (difference was 18.74; *p* < 0.001) had a significant rise from M1 to M2 (min ten minutes, during intervention) (Figure 5a,b). The mean HR change was statistically significant in all the groups, with results inverse to that of mean NN. The maximal significant change was with *raga Malkauns*, where HR reduced by a value of 2.05 bpm (*p* < 0.001) from M1 to M2 and 2.86 bpm (*p* < 0.001) from M1 to M3. The next maximal significant change was observed with *raga Puriya*, with a drop of 1.92 bpm (*p* = 0.01), and *raga Miyan ki Todi*, with a drop of 1.79 bpm (*p* = 0.01) from M1 to M3. The control group had a significant drop from M1 to M2 by about 1.7 bpm (*p* < 0.001) (Table S4, Figure 5c,d). The SDNN change was significant in all the groups. The maximal significant change in SDNN was with *Puriya*, where SDNN increased by 12.24 ms (*p* < 0.001), followed by *raga Miyan ki Todi*, where the level of the rise was 10.03 ms (*p* < 0.001) from M2 to M3. A significant M1 to M3 SDNN increase was seen with *raga Malkauns* (difference of 7.19; *p* = 0.05) and the control group (difference of 9.51; *p* < 0.001) (Figure 5e,f). The mean RMSSD, similar to SDNN, reduced during M2 with *Puriya* and *raga Miyan ki Todi*, but increased beyond baseline during M3; the change was statistically significant in both these groups. The maximal significant change in RMSSD was with *Puriya*, where the RMSSD increased by 9.70 ms (*p* = 0.06), and *Raga Miyan ki Todi*, where the level of the rise was 9.49 ms (*p* = 0.04) from M2 to M3. In the control group, the RMSSD increased significantly from M1 to M3 (7.97 units (*p* = 0.03)) and M2 to M3 (4.36 units (*p* = 0.04)). Group B did not show a significant change in RMSSD (Figure 5g,h). The pNN50 (%) between M1 and M3 was statistically significant only with *raga Puriya* (Figure 5i,j; Table S4, Supplementary File).

##### Frequency-Domain Parameters of Heart Rate Variability

**Figure 6:**
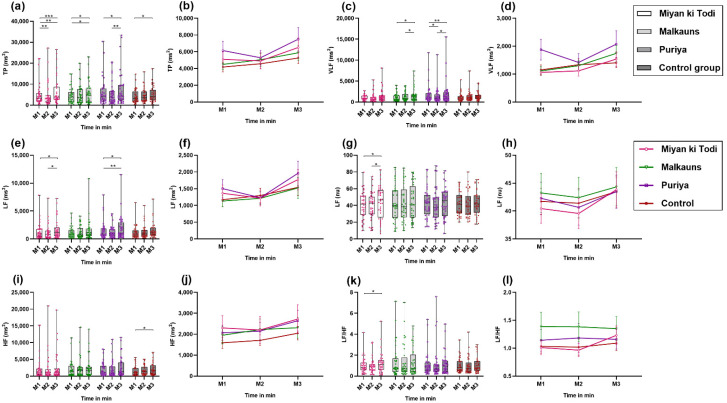
Comparison of frequency-domain parameters of HRV among the four groups at different time points ((M1, M2, M3 is the measurement of HRV pre-intervention (T10), during the intervention (T20), and post-intervention (T30)). (**a**,**b**) TP in ms^2^; (**c**,**d**) VLF in ms^2^; (**e**,**f**) LF in ms^2^; (**g**,**h**) LF in nu; (**i**,**j**) HF in ms^2^; (**k**,**l**) Ratio of LF/HF. Note: * *p* < 0.05, ** *p* < 0.01, *** *p* < 0.001.

In line with the findings of time-domain HRV parameters, TP reduced during M2 with *raga Miyan ki Todi* and *raga Puriya*, and increased beyond baseline levels at M3, while in groups B (*raga Malkauns*) and D (control), TP continuously increased. The maximal significant change in TP (ms^2^) was with *Puriya*, where TP increased by 2211.1 units (*p* = 0.04), followed by *raga Miyan ki Todi*, where the level of the rise was 1597.1 units (*p* = 0.01) from M2 to M3. A significant TP increase was seen from M1 to M3 with *raga Miyan ki Todi* (difference of 1414.3; *p* = 0.01) and *raga Malkauns* (difference of 1379.9; *p* = 0.03) [Figure 6a,b]. There was a significant difference in mean VLF power (ms^2^) with *raga Malkauns* and *raga Puriya* (*p* = 0.013 and 0.007, respectively). The power spectrum of HRV in the VLF range reduced significantly at M2 (by 459.96 units; *p* = 0.05) and tended to increase at M3 (by 652.62 units; *p* = 0.03) with *raga Puriya*. VLF change was also significant, with *raga Malkauns* showing a continuous rise in VLF power, the M1 to M3 difference being statistically significant (630.87 units change; *p* = 0.03) (Figure 6c,d). Like SDNN, RMSSD, TP, the LF in ms^2^ also reduced during M2 with *raga Miyan ki Todi* and *raga Puriya* and increased beyond baseline levels at M3. In that, the change between M2 to M3 was statistically significant with *raga Miyan ki Todi* (rise by 551.22 units; *p* = 0.04), while the change between M1 and M3 was significant with *raga Puriya* (rise of 457.38 units; *p* = 0.01) (Figure 6e,f). The LF (nu) reduced significantly only with *raga Miyan ki Todi* (*p* = 0.014) and increased beyond baseline levels post-intervention (pairwise comparison of M2 to M3, 4.26 units; *p* = 0.03) (Figure 6g,h). A significant rise was seen in HF (ms^2^) only in the control group (*p* = 0.041) (Figure 6i,j). There was a significant change in the LF/HF ratio observed only with *raga Miyan ki Todi* (*p* = 0.028), wherein the LF/HF ratio reduced slightly during the intervention and later increased beyond baseline levels post-intervention (Figure 6k,l; Table S5, Supplementary File).

On univariate analysis of HRV parameters, none of the confounding factors (based on questionnaire data) were found to be associated with the change in the HRV parameters, except VLF ms^2^. Alcoholism history seemed to affect the VLF ms^2^ difference: *F*(1, 131) = 4.844, *p* = 0.029 (log-converted VLF ms^2^ difference *F*(1, 131) = 7.880, *p* = 0.006). It was observed that there was a significant effect of time (M1, M2, M3) on participants who were non-alcoholics (F = 11.315, *p* < 0.0001), compared to alcoholics. Group-wise, no difference was observed. However, the percentage of variation in VLF ms^2^ that could be explained by the independent variables (Supplementary Table S6) was only 9–10%.

## 4. Discussion

In this study, we assessed the effect of passive listening to three different acoustic stimuli (Indian classical music modes) on the cardiovascular electrophysiological effects and subjective behavioral responses (anxiety and stress) among normal healthy individuals and compared them with a control group listening to natural sounds. Three different modes/ragas of Indian classical music used as interventions were *Miyan ki Todi* for group A, *Malkauns* for group B, and *Puriya* for group C. Those in the control arm (group D) relaxed for 30 min while listening to intermittent natural sounds for a very short duration. Sociodemographically, the groups matched, except for educational status, with more graduates or postgraduates in the three intervention groups compared to the control group. All groups matched based on their musical training as well.

### 4.1. Behavioral Analysis

#### Anxiety

As listening to music can initiate a multitude of cognitive processes in the brain [83], it might be assumed that music also influences stress-related cognitive processes and, as a consequence, physiological responses [12]. Anxiety was measured in the current study using a standard validated State-Trait Anxiety Inventory (STAI) Form Y. The three intervention groups showed a significant drop in state anxiety, while the control group had an insignificant mild drop. The maximum reduction in the state score was with *raga* *Puriya*, followed by *raga Malkauns* and *raga Miyan Ki Todi*. This reduction in state anxiety indicated a relaxation response to listening to music. In contrast, trait anxiety increased in all four groups, which could be due to chance or the boredom that set in after answering multiple questions (trait anxiety formed the last 20 questions). A shorter version of the STAI might have been a better tool to assess the trait anxiety after 30–40 min of the protocol. Furthermore, a reduction in trait anxiety might occur after a few weeks or months of music intervention, as we observed previously among pre-hypertensives after listening to *raga Bhimpalas* for 15 min a day, for a minimum of 5 days a week, followed up after 3 months duration [23,38]. In comparison, the three other modes (*raga Ahir Bhairav, Raga Kaunsi Kanada, and Raga Bhimpalas*) reduced the state anxiety levels, with *raga Kaunsi Kanada* causing maximal reduction [22]. The current study’s findings are similar to this, wherein any music intervention reduced anxiety levels, but the level of reduction depended on the melodic mode and, probably, its features. A reduction in anxiety after listening to music is the most consistent finding reported in field studies with patients [84,85,86] and laboratory-based studies [15,84,85]. Music may be a way to help young people reduce negative emotions [15]. Another study suggested listening to relaxing music (based on the music rating scale); in particular, classical music led the listener to experience positive emotions (STAI-Y and Relaxation Rating Scale) and an increase in parasympathetic nervous system arousal (physiological assessment of HR, respiration, and skin conductance) [15].

### 4.2. Biomarkers of Stress

In the current study, we observed that the mean sCort levels reduced in all three music intervention groups during the intervention, but maximally in the control group, which was statistically significant. This is similar to previous research that found lower sCort in the music group when compared to the control groups, but the levels were lowest when participants listened to the sound of rippling water (used as an acoustic control condition) [12]. In the current study, the control group received natural sounds for about 50 s in the mid ten min, indicating that natural sounds have a higher impact on cortisol levels. Several studies have documented either no change or a drop in sCort levels either after listening to music or passive listening to music during a stress task [87,88,89,90]. A decrease in serum cortisol levels was found to be better among men compared to women upon listening to music by Mozart and Strauss [91]. A recent study also observed that the listening environment mattered for this change in cortisol levels or emotions, in that, the cortisol levels were generally lower at home than in the laboratory, though it reduced both in home and lab settings after listening to music [92]. In the current study, the mean sAA levels reduced in all four groups significantly during the intervention, after which the levels increased slightly more than baseline levels. Post hoc analysis showed a significant maximal drop in sAA with *raga Malkauns*, followed by *raga Miyan Ki Todi* and then *raga Puriya*. The rise post-intervention was maximal with *raga Malkauns*, followed by *raga Miyan Ki Todi* and *raga Puriya*. This observation is in line with the HRV changes during and post-intervention, where *Malkauns* exhibited a parasympathetic response during the intervention, while *Miyan Ki Todi* and *Puriya* showed sympathetic responses. A study observed an association between sAA and music-induced arousal, with energizing music increasing and relaxing music decreasing sAA. They proposed that the best effect of music was recorded when participants listened to the music with the intent of ‘relaxation’, which might lead to reduced sAA and sCort levels [47]. In addition to this, another study revealed that listening to Tibetan music before surgery reduced the state score and sAA levels, while in the control group (who wore headphones with no sounds), the state score remained unchanged and the sAA level increased [93].

### 4.3. Cardiovascular Parameters

#### 4.3.1. Blood Pressure

The changes in SBP and DBP were insignificant in all the groups of the present study. A large change in BP was not expected, as all individuals were healthy, aged 18 to 30 years in the current study. Subtle differences in BP were observed in the music intervention groups compared to the control group and also between different modes. Most music-based research has used music as an intervention among hypertensives [23,31], or during or after stressful tasks [94], with very few among healthy individuals in the absence of a task. A meta-analysis showed that music therapy, which is a more intensive intervention than the present study, led to a significant reduction in SBP, DBP, and HR compared to those who did not receive music therapy [29]. Thus, with a longer duration of listening, across multiple days or sessions, differences in SBP and DBP would be evident. Self-selected sedative music induced both aroused and sedative emotions and a slight but significant increase in HR [95]. In the present study, participants were not given a choice to select their music, or their preference was not taken into account during the planning of the intervention. In addition, continuous monitoring of BP fluctuations might have aided us to obtain more conclusive results.

#### 4.3.2. Heart Rate Variability

In the present study, we found that the mean NN interval increased and the mean HR reduced significantly in all four groups. The maximal significant change in HR was with *raga Malkauns*. When looking at the time and frequency-domain measurements we observed that *raga Miyan ki Todi* (group A) and *Puriya* (group C) caused arousal effects in the form of a drop of SDNN, RMSSD, TP (ms^2^), VLF (ms^2^), LF (ms^2^), and HF (ms^2^) during the intervention, with significant relaxation after the intervention was stopped. SDNN and RMSSD are strong indicators of parasympathetic activity [37,96]. This shows that *raga Miyan ki Todi* and *Puriya* caused sympathetic arousal during music while increasing the parasympathetic response after the music has stopped. This seems similar to previous studies showing increased sympathetic activity, regardless of the type of music (calming or stimulating) [46,97], and a classic paper by Bernardi et al., where a pause after playing music for 2 min exhibited the maximal relaxation response [40]. In contrast, the *raga Malkauns* results went hand-in-hand with the control group, wherein a sustained increase in parasympathetic response was observed over 30 min. These findings are partly in sync with the BP results quoted above, suggesting that the changes observed between the cardiovascular and the autonomic system were in parallel. A recent review observed that, out of 29 randomized trials and pre to post-intervention studies, 26 studies suggested a significant positive impact of music on HRV [50]. In a study where *raga Malkauns* was used as an intervention in different forms (vocal rendition, sitar recital, and *Rabindra sangeet*), *Rabindra sangeet* had the most relaxing effect. In addition, *alaap* delivered at a fast tempo increased excitement, while, like the current study, *alaap* at a slow tempo resulted in calming the mood [56]. Unlike in the current study, meditative music has been shown to reduce state anxiety and HR, and increase the HF norm of HRV [98]. These results also confirm the importance of analysis of temporal changes in physiological parameters when using music as an intervention, as the music unfolds over time [99]. Other studies on HRV using music intervention have been detailed before in [38,50]. Regarding the mechanism behind the effect of auditory stimulation and cardiac autonomic regulation, it was hypothesized that pleasurable songs induce dopamine release in the striatal system, which is involved in autonomic regulation, and this topic has been well-reviewed in [100]. 

### 4.4. Future Directions

The current study used a triple-blinded, randomized control trial design and showed that listening to three different Indian modes caused behavioral and cardiovascular modifications among healthy adults. This is the first study of its kind to focus on how Indian melodies may alter physiological measures related to stress, arousal, and anxiety. Clinically, this study promotes the idea of the use of music, and particular modes, to facilitate relaxation, prevent cardiovascular disease, and provide an alternative treatment strategy. Future studies may find it beneficial to expand the present findings to other melodies, provide longer periods of music listening, and more closely investigate in both males and females how reproductive steroid hormones may play a role in the physiological measures assessed. It would also be interesting to investigate factors related to perception and emotion, such as personality and music preferences, in future work. Further analysis of the musical features and the components (e.g., temporal analysis of note/tonal variations, pitch, tempo, dynamics, contrast) of the music used may enhance our understanding of the physiological effects.

## 5. Conclusions

Among the different relaxation therapies known to us, music is an important modality, as it is an easy-to-follow, easy-to-use, inexpensive mode of relaxation. This study provides evidence that listening to music for just 10 min can have an acute reduction in anxiety and improvement in cardiovascular parameters, depending on the mode. Future studies may try to elucidate the role of music after intervention over a longer duration or a few months of intervention, as done in [1,2]. Though all three modes (*ragas*) reduced state anxiety scores, *raga Puriya* caused a maximal reduction in state anxiety scores, followed by *Malkauns* and *Miyan ki Todi.* Cardiovascular effects went in hand with the behavioral recordings, in that *raga Puriya* and *raga Miyan ki Todi* produced an arousal effect during music intervention but caused significant relaxation after the intervention was stopped. In contrast, *raga Malkauns* reduced state anxiety, significantly increased the mean NN interval, and reduced HR. This proves that listening to music, in general, cannot be said to produce a relaxation effect; rather, the timing of the effect, the notes/tones present in the music given and the combination of notes (modes) as a whole that produces a particular effect. Future studies need to emphasize the health benefits of various aspects of different acoustic stimuli, including other types and genres of music, and establish solid evidence for the usage of the same in different medical disorders.

## Figures and Tables

**Table 1:** The three chosen Indian melodic modes, the names of the notes in Hindustani music, and Western scale equivalents.

Svara/Note	Hindustani Name	Staff Note	Western Scale Interval Name
	***Raga Miyan ki Todi (Scale A) (heptatonic, G appears in descent)***		
S	*Shadja*	C	Perfect unison
r	*Komal Rishab*	D♭	Minor second
g	*Komal Gandhar*	E♭	Minor third
M	*Tivra Madhyam*	F#	Augmented fourth
P	*Pancham*	G	Perfect fifth
d	*Komal Dhaivat*	A♭	Minor sixth
N	*Shuddha Nishad*	B	Major seventh
	***Raga Malkauns (Scale B) Ascent and descent same—pentatonic***		
S	*Shadja*	C	Perfect unison
g	*Komal Gandhar*	E♭	Minor third
m	*Shuddha Madhyam*	F	Perfect fourth
d	*Komal Dhaivat*	A♭	Minor sixth
n	*Komal Nishad*	B♭	Minor seventh
	***Raga Puriya (Scale C) C, D* *♭* *, E, G* *♭* *, G, A/A* *♭* *, B (hexatonic)***		
S	*Shadja*	C	Perfect unison
r	*Komal Rishab*	D♭	Minor second
G	*Shuddha Gandhar*	E	Major third
M	*Tivra Madhyam*	F#	Augmented fourth
D	*Shuddha Dhaivat*	A	Major sixth
N	*Shuddha Nishad*	B	Major seventh

**Table 2:** Sociodemographic characteristics of participants.

Variables	Group A	Group B	Group C	Group D	*p*-Value
Sample	N = 37 (%)	N = 36 (%)	N = 36 (%)	N = 35 (%)	
**Age (Years)**					
<=18	9 (24.3)	5 (13.9)	6 (16.7)	4 (11.4)	0.171
19–21	18 (48.6)	18 (50.0)	15 (41.7)	19 (54.3)	
22–24	8 (21.6)	11 (30.6)	11 (30.6)	4 (11.4)	
>=25	2 (5.4)	2 (5.6)	4 (11.1)	8 (22.9)	
**Age (years) Mean, SD**	20.54, 2.5	20.75, 2.5	21.11, 2.6	21.26, 3.0	0.646
**Gender**					
Female	29 (78.4)	20 (55.6)	24 (66.7)	25 (71.4)	0.202
Male	8 (21.6)	16 (44.4)	12 (33.3)	10 (28.6)	
**Education**					
High school/Intermediate	16 (43.2)	7 (19.4)	16 (44.4)	29 (82.9)	<0.001
Graduate/Postgraduate	21 (56.8)	29 (80.6)	20 (55.6)	6 (17.1)	
**Marital status**					
Married	36 (97.3)	35 (97.2)	35 (97.2)	33 (94.3)	0.875
Single	1 (2.7)	1 (2.8)	1 (2.8)	2 (5.7)	
**Diet**					
Vegetarian	14 (37.8)	11 (30.6)	7 (19.4)	16 (45.7)	0.112
Non-vegetarian	23 (62.2)	25 (69.4)	29 (80.6)	19 (54.3)	
**BMI (kg/m^2^) Mean, SD**	23.17, 3.96	22.96, 4.71	22.16, 3.47	22.47, 4.10	0.714
**Music Training—Yes—N (%)**	17 (45.9)	14 (38.9)	11 (30.6)	12 (34.3)	0.562

**Note:** N is the number of participants in each group; All the values of the two groups are in absolute values and parenthesis contain percentages; a *p*-Value of <0.05 is considered significant; P calculated using Chi-square test/Fisher exact test; Mean age and BMI comparison was done using ANOVA.

**Table 3:** Comparison of pre and post-intervention STAI scores between four groups.

Group		Mean	SD	md	Quartiles			*p*
					25	50	75	
**STAI State Anxiety**								
**A (N = 37)**	Pre	35.16	10.8	2.35	28.5	33	40	0.054
	Post	32.81	10.7		24.5	30	39	
**B (N = 36)**	Pre	34.92	12.3	3.83	24.5	30.5	44.3	0.057
	Post	31.08	8.9		23.5	29	34	
**C (N = 36)**	Pre	36.11	11.7	3.94	25.5	35.5	43.8	0.018
	Post	32.17	10.3		25	29.5	37	
**D (N = 34)**	Pre	34.21	7.2	0.32	30	32.5	37	0.781
	Post	33.74	8.3		28	30.5	40	
**STAI Trait Anxiety**								
**A (N = 37)**	Pre	56	10.4	−1.87	47	58	63.5	0.057
	Post	57.87	9.8		51	58	64	
**B (N = 36)**	Pre	57.44	8.4	−1.08	51.25	57	64	0.135
	Post	58.53	8.2		53.3	59	63	
**C (N = 36)**	Pre	57.69	9.3	−2.33	51	57.5	64.5	0.011
	Post	60.03	9.4		53	60	66.8	
**D (N = 34)**	Pre	59.32	8.0	−0.88	53	60	65	0.302
	Post	60.09	9.0		54	61	67	

Note: *p* value < 0.05 was considered significant, calculated using paired *t*-test.

## Data Availability

The data presented in this study are available on request from the corresponding author. The data are not publicly available due to ethical reasons.

## Supplementary Materials

The following supporting information can be downloaded at: https://www.mdpi.com/article/10.3390/ejihpe12100108/s1 [101,102,103,104,105].

## References

[1] Schäfer T., Tipandjan A., Sedlmeier P. (2012). The functions of music and their relationship to music preference in India and Germany. Int. J. Psychol..

[2] Montinari M.R., Giardina S., Minelli P., Minelli S. (2018). History of Music Therapy and Its Contemporary Applications in Cardiovascular Diseases. South. Med. J..

[3] Definition and Quotes about Music Therapy | Definition and Quotes about Music Therapy | American Music Therapy Association (AMTA). https://www.musictherapy.org/.

[4] McCrary J.M., Redding E., Altenmüller E. (2021). Performing arts as a health resource? An umbrella review of the health impacts of music and dance participation. PLoS ONE.

[5] Fancourt D., Finn S. (2019). What Is the Evidence on the Role of the Arts in Improving Health and Well-Being. A Scoping Review.

[6] Reybrouck M., Vuust P., Brattico E. (2021). Neural Correlates of Music Listening: Does the Music Matter?. Brain Sci..

[7] Engel A., Hoefle S., Monteiro M.C., Moll J., Keller P.E. (2022). Neural Correlates of Listening to Varying Synchrony Between Beats in Samba Percussion and Relations to Feeling the Groove. Front. Neurosci..

[8] Biagini M.S., Brown L.E., Coburn J.W., Judelson D.A., Statler T.A., Bottaro M., Tran T.T., Longo N.A. (2012). Effects of Self-Selected Music on Strength, Explosiveness, and Mood. J. Strength Cond. Res..

[9] Vinciguerra C., De Stefano N., Federico A. (2019). Exploring the role of music therapy in multiple sclerosis: Brief updates from research to clinical practice. Neurol. Sci..

[10] Nardone V., Vinciguerra C., Correale P., Guida C., Tini P., Reginelli A., Cappabianca S. (2020). Music therapy and radiation oncology: State of art and future directions. Complement. Ther. Clin. Pract..

[11] Linnemann A., Wenzel M., Grammes J., Kubiak T., Nater U.M. (2018). Music Listening and Stress in Daily Life—A Matter of Timing. Int. J. Behav. Med..

[12] Thoma M.V., La Marca R., Brönnimann R., Finkel L., Ehlert U., Nater U.M. (2013). The Effect of Music on the Human Stress Response. PLoS ONE.

[13] Linnemann A., Strahler J., Nater U.M. (2016). The stress-reducing effect of music listening varies depending on the social context. Psychoneuroendocrinology.

[14] Linnemann A., Strahler J., Nater U.M. (2017). Assessing the Effects of Music Listening on Psychobiological Stress in Daily Life. J. Vis. Exp..

[15] Labbe E., Schmidt N., Babin J., Pharr M. (2007). Coping with Stress: The Effectiveness of Different Types of Music. Appl. Psychophysiol. Biofeedback.

[16] Alagha J., Ipradjian A. (2017). The Effects of Different Types of Music on Stress Levels. Glob. J. Hum.-Soc. Sci. A Arts Humanit.—Psychol..

[17] De Witte M., Spruit A., Van Hooren S., Moonen X., Stams G.-J. (2020). Effects of music interventions on stress-related outcomes: A systematic review and two meta-analyses. Health Psychol. Rev..

[18] Wuttke-Linnemann A., Nater U.M., Ehlert U., Ditzen B. (2019). Sex-specific Effects of Music Listening on Couples’ Stress in Everyday Life. Sci. Rep..

[19] Pezzin L.E., Larson E.R., Lorber W., McGinley E.L., Dillingham T.R. (2018). Music-instruction intervention for treatment of post-traumatic stress disorder: A randomized pilot study. BMC Psychol..

[20] Hegde S. (2017). Music therapy for mental disorder and mental health: The untapped potential of Indian classical music. BJPsych. Int..

[21] Knobloch S., Zillmann D. (2002). Mood Management via the Digital Jukebox. J. Commun..

[22] Ubrangala K.K., Kunnavil R., Goturu J., Vijayadas M., Prakash V.S., Murthy N.S. (2021). Effect of specific melodic scales of Indian music in reducing state and trait anxiety: A randomized clinical trial. Psychol. Music.

[23] Kunikullaya K.U., Goturu J., Muradi V., Hukkeri P.A., Kunnavil R., Doreswamy V., Prakash V.S., Murthy N.S. (2016). Combination of music with lifestyle modification versus lifestyle modification alone on blood pressure reduction—A randomized controlled trial. Complement. Ther. Clin. Pract..

[24] Lu G., Jia R., Liang D., Yu J., Wu Z., Chen C. (2021). Effects of music therapy on anxiety: A meta-analysis of randomized controlled trials. Psychiatry Res..

[25] Kühlmann A.Y.R., de Rooij A., Kroese L.F., van Dijk M., Hunink M.G.M., Jeekel J. (2018). Meta-analysis evaluating music interventions for anxiety and pain in surgery. Br. J. Surg..

[26] Van Willenswaard K.C., Lynn F., McNeill J., McQueen K., Dennis C.-L., Lobel M., Alderdice F. (2017). Music interventions to reduce stress and anxiety in pregnancy: A systematic review and meta-analysis. BMC Psychiatry.

[27] Márquez-Celedonio F.G., Téxon-Fernández O., Chávez-Negrete A., Hernández-López S., Marín-Rendónm S., Berlín-Lascurain S. (2009). Clinical effect of lifestyle modification on cardiovascular risk in prehypertensives: PREHIPER I study. Rev. Esp. Cardiol..

[28] Bekiroğlu T., Ovayolu N., Ergün Y., Ekerbiçer H. (2013). Effect of Turkish classical music on blood pressure: A randomized controlled trial in hypertensive elderly patients. Complement. Ther. Med..

[29] Loomba R.S., Arora R., Shah P.H., Chandrasekar S., Molnar J. (2012). Effects of music on systolic blood pressure, diastolic blood pressure, and heart rate: A meta-analysis. Indian Heart J..

[30] Im-Oun S., Kotruchin P., Thinsug P., Mitsungnern T., Techa-Atik P., Pongchaiyakul C. (2018). Effect of Thai instrumental folk music on blood pressure: A randomized controlled trial in stage-2 hypertensive patients. Complement. Ther. Med..

[31] Amaral M.A.S.D., Neto M.G., de Queiroz J.G., Martins-Filho P.R.S., Saquetto M.B., Carvalho V.O. (2016). Effect of music therapy on blood pressure of individuals with hypertension: A systematic review and Meta-analysis. Int. J. Cardiol..

[32] Salmore R.G., Nelson J.P. (2000). The effect of preprocedure teaching, relaxation instruction, and music on anxiety as measured by blood pressures in an outpatient gastrointestinal endoscopy laboratory. Gastroenterol. Nurs..

[33] Smolen D., Topp R., Singer L. (2002). The effect of self-selected music during colonoscopy on anxiety, heart rate, and blood pressure. Appl. Nurs. Res..

[34] Dileo C. (2006). Effects of music and music therapy on medical patients: A meta-analysis of the research and implications for the future. J. Soc. Integr. Oncol..

[35] Cadigan M.E., Caruso N.A., Haldeman S.M., McNamara M.E., Noyes D.A., Spadafora M.A., Carroll D.L. (2001). The Effects of Music on Cardiac Patients on Bed Rest. Prog. Cardiovasc. Nurs..

[36] Berbel P., Moix J., Quintana S. (2007). Music versus diazepam to reduce preoperative anxiety: A randomized controlled clinical trial. Rev. Esp. Anestesiol. Reanim..

[37] Kunikullaya U.K., Kunnavil R., Vijayadas, Goturu J., Prakash V.S., Murthy N.S. (2021). Normative data and gender differences in heart rate variability in the healthy young individuals aged 18–30 years, a South Indian cross-sectional study. Indian Pacing Electrophysiol. J..

[38] Kunikullaya K.U., Goturu J., Muradi V., Hukkeri P.A., Kunnavil R., Doreswamy V., Prakash V.S., Murthy N.S. (2015). Music versus lifestyle on the autonomic nervous system of prehypertensives and hypertensives—A randomized control trial. Complement. Ther. Med..

[39] Hamel W.J. (2001). The effects of music intervention on anxiety in the patient waiting for cardiac catheterization. Intensive Crit. Care Nurs..

[40] Bernardi L., Porta C., Sleight P. (2006). Cardiovascular, cerebrovascular, and respiratory changes induced by different types of music in musicians and non-musicians: The importance of silence. Heart.

[41] Larsen P.D. (2006). The sound of silence is music to the heart. Heart.

[42] Okada K., Kurita A., Takase B., Otsuka T., Kodani E., Kusama Y., Atarashi H., Mizuno K. (2009). Effects of music therapy on autonomic nervous system activity, incidence of heart failure events, and plasma cytokine and catecholamine levels in elderly patients with cerebrovascular disease and dementia. Int. Heart J..

[43] Yamamoto T., Ohkuwa T., Itoh H., Kitoh M., Terasawa J., Tsuda T., Kitagawa S., Sato Y. (2003). Effects of Pre-exercise Listening to Slow and Fast Rhythm Music on Supramaximal Cycle Performance and Selected Metabolic Variables. Arch. Physiol. Biochem..

[44] Urakawa K., Yokoyama K. (2005). Music Can Enhance Exercise-Induced Sympathetic Dominancy Assessed by Heart Rate Variability. Tohoku J. Exp. Med..

[45] Dey A., Bhattacharya D.K., Tibarewala D.N., Palit S.K. (2013). Effect of Music on Autonomic Nervous System through the Study of Symbolic Dynamics of Heart Rate Variability Signals. J. Proc..

[46] Shimomura Y., Hoshiba K., Morigiwa T., Matsumoto K. (1997). Psychophysiological study of music stimuli on music therapy. J. Jpn. Biomusic. Assoc..

[47] Linnemann A., Ditzen B., Strahler J., Doerr J.M., Nater U.M. (2015). Music listening as a means of stress reduction in daily life. Psychoneuroendocrinology.

[48] Lieber A.C., Bose J., Zhang X., Seltzberg H., Loewy J., Rossetti A., Mocco J., Kellner C.P. (2019). Effects of music therapy on anxiety and physiologic parameters in angiography: A systematic review and meta-analysis. J. Neurointerv. Surg..

[49] Panteleeva Y., Ceschi G., Glowinski D., Courvoisier D.S., Grandjean D. (2018). Music for anxiety? Meta-analysis of anxiety reduction in non-clinical samples. Psychol. Music.

[50] Mojtabavi H., Saghazadeh A., Valenti V.E., Rezaei N. (2020). Can music influence cardiac autonomic system? A systematic review and narrative synthesis to evaluate its impact on heart rate variability. Complement. Ther. Clin. Pract..

[51] Kühlmann A.Y.R., Etnel J.R.G., Roos-Hesselink J.W., Jeekel J., Bogers A.J.J.C., Takkenberg J.J.M. (2016). Systematic review and meta-analysis of music interventions in hypertension treatment: A quest for answers. BMC Cardiovasc. Disord..

[52] Mathur A., Vijayakumar S.H., Chakrabarti B., Singh N.C. (2015). Emotional responses to Hindustani raga music: The role of musical structure. Front. Psychol..

[53] Midya V., Valla J., Balasubramanian H., Mathur A., Singh N.C. (2019). Cultural differences in the use of acoustic cues for musical emotion experience. PLoS ONE.

[54] Bowling D.L., Sundararajan J., Han S., Purves D. (2012). Expression of Emotion in Eastern and Western Music Mirrors Vocalization. PLoS ONE.

[55] Balkwill L.-L., Thompson W. (1999). A Cross-Cultural Investigation of the Perception of Emotion in Music: Psychophysical and Cultural Cues. Music Percept..

[56] Mukherjee S., Palit S.K., Banerjee S., Bhattacharya D.K. (2015). A Comparative Study on Three Different Types of Music Based on Same Indian Raga and Their Effects on Human Autonomic Nervous Systems. Chaos, Complexity and Leadership 2013.

[57] Osmer B. ‘Raga Chikitsa and Raga Ragini Vidya—Bill Osmer’, December 2006. pp. 1–29. https://swaraabhimanee.files.wordpress.com/2016/11/raga-ragani-vidya.pdf.

[58] Jairazbhoy N.A. (1995). The Rāgs of North Indian Music: Their Structure and Evolution.

[59] ITC Sangeet Research Academy. https://www.itcsra.org/cronology600.

[60] Kaufmann W. (1965). Rasa, Raga-Mala and Performance Times in North Indian Ragas. Ethnomusicology.

[61] McNeil A. Ragas, Recipes, and Rasas, Oxford Handbooks Online, 7 April 2015. https://www.oxfordhandbooks.com/view/10.1093/oxfordhb/9780199935321.001.0001/oxfordhb-9780199935321-e-43.

[62] WMA—The World Medical Association-WMA Declaration of Helsinki—Ethical Principles for Medical Research Involving Human Subjects. https://www.wma.net/policies-post/wma-declaration-of-helsinki-ethical-principles-for-medical-research-involving-human-subjects/.

[63] Graff V., Cai L., Badiola I., Elkassabany N.M. (2019). Music versus midazolam during preoperative nerve block placements: A prospective randomized controlled study. Reg. Anesth. Pain Med..

[64] Idrobo-Ávila E.H., Loaiza-Correa H., Van Noorden L., Muñoz-Bolaños F.G., Vargas-Canas R. (2018). Different Types of Sounds and Their Relationship with the Electrocardiographic Signals and the Cardiovascular System—Review. Front. Physiol..

[65] McNeil A. (2017). Seed ideas and creativity in Hindustani raga music: Beyond the composition–improvisation dialectic. Ethnomusicol. Forum.

[66] Bansuri, Wikipedia. 30 March 2018. https://en.wikipedia.org/w/index.php?title=Bansuri&oldid=833200245.

[67] Watanabe K., Ooishi Y., Kashino M. (2017). Heart rate responses induced by acoustic tempo and its interaction with basal heart rate. Sci. Rep..

[68] Liu Y., Liu G., Wei D., Li Q., Yuan G., Wu S., Wang G., Zhao X. (2018). Effects of Musical Tempo on Musicians’ and Non-musicians’ Emotional Experience When Listening to Music. Front. Psychol..

[69] Levitin D.J., Grahn J.A., London J. (2018). The Psychology of Music: Rhythm and Movement. Annu. Rev. Psychol..

[70] Zhao T.C., Lam H.G., Sohi H., Kuhl P.K. (2017). Neural processing of musical meter in musicians and non-musicians. Neuropsychologia.

[71] Stith C.C. (2015). The Effects of Musical Tempo and Dynamic Range on Heart Rate Variability in Healthy Adults: A Counterbalanced, Within-subjects Study. Ph.D. Thesis.

[72] Patel A.D., Iversen J. (2014). The evolutionary neuroscience of musical beat perception: The Action Simulation for Auditory Prediction (ASAP) hypothesis. Front. Syst. Neurosci..

[73] Daly I., Hallowell J., Hwang F., Kirke A., Malik A., Roesch E., Weaver J., Williams D., Miranda E., Nasuto S.J. Changes in music tempo entrain movement related brain activity. Proceedings of the 2014 36th Annual International Conference of the IEEE Engineering in Medicine and Biology Society.

[74] Schaub K., Demos L., Centeno T., Daugherty B. (2011). Effects of Musical Tempo on Heart Rate, Brain Activity, and Short-term Memory. J. Adv. Stud. Sci..

[75] State-Trait Anxiety Inventory for Adults (STAI-AD)-Assessments, Tests | Mind Garden-Mind Garden. https://www.mindgarden.com/145-state-trait-anxiety-inventory-for-adults.

[76] Julian L.J. (2011). Measures of anxiety: State-Trait Anxiety Inventory (STAI), Beck Anxiety Inventory (BAI), and Hospital Anxiety and Depression Scale-Anxiety (HADS-A). Arthritis Care Res..

[77] Grös D.F., Antony M.M., Simms L.J., McCabe R.E. (2007). Psychometric properties of the State-Trait Inventory for Cognitive and Somatic Anxiety (STICSA): Comparison to the State-Trait Anxiety Inventory (STAI). Psychol. Assess..

[78] Riquelme A.G., Buela-Casal G. (2014). Meta-analysis of group comparison and meta-analysis of reliability generalization of the State-Trait Anxiety Inventory Questionnaire (STAI). Rev. Española Salud Pública.

[79] Petrakova L., Doering B.K., Vits S., Engler H., Rief W., Schedlowski M., Grigoleit J.-S. (2015). Psychosocial Stress Increases Salivary Alpha-Amylase Activity Independently from Plasma Noradrenaline Levels. PLoS ONE.

[80] Nater U.M., Abbruzzese E., Krebs M., Ehlert U. (2006). Sex differences in emotional and psychophysiological responses to musical stimuli. Int. J. Psychophysiol..

[81] Skosnik P.D., Chatterton R.T., Swisher T., Park S. (2000). Modulation of attentional inhibition by norepinephrine and cortisol after psychological stress. Int. J. Psychophysiol..

[82] Takahashi H., Yoshika M., Yokoi T. (2015). Validation of three automatic devices for the self-measurement of blood pressure according to the European Society of Hypertension International Protocol revision 2010: The Omron HEM-7130, HEM-7320F, and HEM-7500F. Blood Press. Monit..

[83] Peretz I., Zatorre R.J. (2005). Brain Organization for Music Processing. Annu. Rev. Psychol..

[84] Ventura T., Gomes M., Carreira T. (2012). Cortisol and anxiety response to a relaxing intervention on pregnant women awaiting amniocentesis. Psychoneuroendocrinology.

[85] Wang S.-M., Kulkarni L., Dolev J., Kain Z.N. (2002). Music and preoperative anxiety: A randomized, controlled study. Anesth. Analg..

[86] Voss J.A., Good M., Yates B., Baun M.M., Thompson A., Hertzog M. (2004). Sedative music reduces anxiety and pain during chair rest after open-heart surgery. Pain.

[87] Khalfa S., Bella S.D., Roy M., Peretz I., Lupien S.J. (2003). Effects of Relaxing Music on Salivary Cortisol Level after Psychological Stress. Ann. N. Y. Acad. Sci..

[88] Uedo N., Ishikawa H., Morimoto K., Ishihara R., Narahara H., Akedo I., Ioka T., Kaji I., Fukuda S. (2004). Reduction in salivary cortisol level by music therapy during colonoscopic examination. Hepatogastroenterology.

[89] Hasanah I., Mulatsih S., Haryanti F., Haikal Z. (2020). Effect of music therapy on cortisol as a stress biomarker in children undergoing IV-line insertion. J. Taibah Univ. Med. Sci..

[90] Hou Y.-C., Lin Y.-J., Lu K.-C., Chiang H.-S., Chang C.-C., Yang L.-K. (2017). Music Therapy-Induced Changes in Salivary Cortisol Level are Predictive of Cardiovascular Mortality in Patients under Maintenance Hemodialysis. Ther. Clin. Risk Manag..

[91] Trappe H.-J., Voit G. (2016). The Cardiovascular Effect of Musical Genres. Dtsch. Arztebl. Int..

[92] Tervaniemi M., Makkonen T., Nie P. (2021). Psychological and Physiological Signatures of Music Listening in Different Listening Environments—An Exploratory Study. Brain Sci..

[93] Cotoia A., Dibello F., Moscatelli F., Sciusco A., Polito P., Modolo A., Gallo C., Cibelli G., Cinnella G. (2018). Effects of Tibetan Music on Neuroendocrine and Autonomic Functions in Patients Waiting for Surgery: A Randomized, Controlled Study. Anesthesiol. Res. Pract..

[94] Chafin S., Roy M., Gerin W., Christenfeld N. (2004). Music can facilitate blood pressure recovery from stress. Br. J. Health Psychol..

[95] Lingham J., Theorell T. (2009). Self-selected “favourite” stimulative and sedative music listening—How does familiar and preferred music listening affect the body?. Nord. J. Music Ther..

[96] Shaffer F., Ginsberg J.P. (2017). An Overview of Heart Rate Variability Metrics and Norms. Front. Public Health.

[97] Kunikullaya U.K., Vijayadas, Kunnavil R., Goturu J., Prakash V.S., Murthy N.S. (2022). Short-term effects of passive listening to an Indian musical scale on blood pressure and heart rate variability among healthy individuals—A randomised controlled trial. Indian J. Physiol. Pharmacol..

[98] Lee W.-L., Sung H.-C., Liu S.-H., Chang S.-M. (2017). Meditative music listening to reduce state anxiety in patients during the uptake phase before positron emission tomography (PET) scans. Br. J. Radiol..

[99] Kirthana Kunikullaya U., Sasidharan A., Srinivasa R., Goturu J., Murthy N.S. (2022). Temporal changes in electroencephalographic power spectrum on passive listening to three selected melodic scales of Indian music on healthy young individuals—A randomized controlled trial. Music. Med..

[100] Valenti V.E., Guida H.L., Frizzo A.C.F., Cardoso A.C.V., Vanderlei L.C.M., de Abreu L.C. (2012). Auditory stimulation and cardiac autonomic regulation. Clinics.

[101] Raja D. ‘Deepak Raja’s World of Hindustani Music: Raga Miya-Ki Todi…. Reluctant Differentiation’, *Deepak Raja’s World of Hindustani Music*, 24 April 2011. http://swaratala.blogspot.com/2011/04/raga-miya-ki-todi-reluctant.html.

[102] Film Songs in Rag Mian Ki Todi. https://chandrakantha.com/raga_raag/film_song_raga/mian_ki_todi.shtml.

[103] ‘The Raga Guide—Malkauns’, 20 June 2009. https://web.archive.org/web/20090620030217/http://www.wyastone.co.uk/nrl/world/raga/malkauns.html.

[104] Raag Puriya—Indian Classical Music—Tanarang.com. http://www.tanarang.com/english/puriya_eng.htm.

[105] ‘Puriya’, *Wikipedia*. 20 November 2019. https://en.wikipedia.org/w/index.php?title=Puriya&oldid=927072231.

