# Supplementary material for: Effect of Indian Music as an Auditory Stimulus on Physiological Measures of Stress, Anxiety, Cardiovascular and Autonomic Responses in Humans—A Randomized Controlled Trial
Source: Eur J Investig Health Psychol Educ. 2022 Oct 19;12(10):1535–58. doi: 10.3390/ejihpe12100108 (PMC9601678; doi:10.3390/ejihpe12100108)
Supplement: Supplementary file 1 [file ejihpe-12-00108-s001.zip › ejihpe-1793486-supplementary.pdf]

S1. Music intervention—Melodic scales in detail

***Miyan ki Todi, raga* A of this study,** is a Hindustani classical raga that gave its name to the *Todi thaat,* one of the ten modes of Hindustani classical music, also known as *Darbari Todi,* and sometimes *Shuddha Todi;* it is amongst the more popular morning *raga*s of Hindustani music. The scale of *Miyan ki Todi* is *Arohana:* S r g m d N S' or 'd 'N S r g m d N S' or S r g m d P, m d N S' or S r g m P, m d N S' and *Avarohana:* S' N d P m g r S or S' N d P m d m g r g r S. *Vadi* and *Samavadi* are *Komal Dha* and *Komal Ga*. *Re, ga,* and *dha* are intoned slightly low, and *tivra ma* is very sharp. Bhatkhande pronounces *Komal Dha* as *Vadi* (primarily dominant), but some musicians accord this status to *Komal Ga*. According to him, *Komal Ga* and *Komal Re* are candidates for the status of *samvadi* (secondary dominant). *Todi* is a *raga* of the late morning. The prescribed time for the *raga* is the first three-hour slot after sunrise. The equivalent *raga* in Carnatic music is *Shubhapantuvarali. Miyan ki Todi* predominantly is mostly pervaded by a pensive, mournful mood, which is then relieved in the *drut* (faster tempo) part by a festive piece, possibly to alleviate the heavy pathos in the earlier stages of rendering, though not always. The composition is such as to afford an artist of high caliber to mold it in either the inherent pensive mood or to entirely present a festive mood. Despite this, the *raga* has attained a decent presence in the classicist, as well as romanticist, genres of Hindustani music. The common phrases used in this scale are: N N S r g / r g r / r g M^ P or r g M^ g P/ g M^ d P / M^ g M^ d / N d P / d d N S′ [or] M^ d N S′/ N S′ r′ g′ r′/ d N S′ r′ g′ / r′ g′ r′ S′/ N r′ N d P / M^ P d M^ g [or] N d M^ g / r g r S [1]. Popular songs based on this *raga* are: *Bhini bhini bhor (Asha Bhosle's Album Dil Padosi Hai), aeri mai to prem diwani mera dard na jane koi (A meerabai bhajan from the movie Meera), Watan pe jo fida hoga (Phool bane angaare* movie) [2].

Raga Malkauns belongs to *Kalyan thaat* and is a majestic and somewhat introverted pentatonic *raga*. *Ma* is the pivotal tone of this *raga* and the tone in which the first string of the *tanpura* is usually tuned. *Ga, Dha,* and *Ni* may slightly oscillate. *Malkauns* should be performed in a slow and dignified manner, and to bring out its ethos the notes should be linked by glides, in particular N / D, D / M, and M / G [3]. Time: Late night, 12 - 3. *Aarohan* (ascending scale): S G M D N M D S*; *Avaraohan* (descending scale): S* N D M G M G S, D S; * indicates a higher (third) octave. The *Rishabh* and the *pancham* are skipped in the scale. It is an *audhav–audhav* (five notes in ascent and descent of the scale) *vakra (nishad* is rarely employed in *avaroh)*. The *vaadi samavaadi swaras* for this *raga* are d and g. The *vishranti sthaan* for this scale are G; D; S'; - D; G. Example of *sanchar* (move/phrases/flow) through this *raga,* S; G M D G M G ; M G ; G S ; ,D ,D S ; ,N ,M ,D S ; S G M D ; G M G ; M D S' ; N M D ; G M M G ; S ; ,D ,D S. It is this preponderance of the *tivra madhyam* that requires intense training to perform this *raga*. Time for best effect is between 12 am and 3 am: 3rd *prahar* of the night (*ragas* are divided into *prahaars* whereby each *raga* has a specific period of the day when it is performed). The popular Hindi film songs based on *raga Malkauns* include *Aaye Sur Ke Panchhi Aaye (Sur Sangam* movie*), Adha Hai Chandrama Raat Adhi* and *Tu Chhupi Hai Kahan (Navrang), Man Tarapat Hari Darshan Ko (Baiju Bawra)* [3]. **Malkauns was *Raga* B in this study.**

Raga Puriya is a major hexatonic *raga* (*Shadhav–Shadhav*) of Hindustani classical music, belonging to the *marwa thaat*. Best performed just after sunset (2nd *prahar* of the night). What is common among all types of *Puriya raag* are *komal* (flat) *Re, shuddha* (natural) *Ga, tivra* (sharp) *Ma,* and *shuddha* (natural) *Ni. Aarohan*: N r G M D N r S and *avarohan*: S N D M G r S N or r N D M Gg, M G r S. *Pancham Varjya. Rishabh Komal, Madhyam Teevra*. Rest all *Shuddha Swaras*. Mandra Saptak Nishad is the Nyas swar in Puriya. Illustrative combinations are: N r G ; G r ,N ,D ,N; ,N ,M ,D S; G M D N; N M G; G M D G M G; r S; G M D N D S'; N r' N M G ; G M D G M G r S [4]. In this *raga,* N–M and D–G *sangati* is observed. Nishad is often skipped in *Aaroh* like G M D N D S'. *Raag Puriya* is often referred to as king of the night *ragas*. The *rasa* / emotions related to this *raga* are *Shanti* (equanimity/peace) and *Gambhir* (seriousness) [5]. ***Puriya* was *Raga* C in this study.** Pure *Puriya* has not been very commonly used for film music.

S2. Music Exposure and Training:

Of the participants who were trained, 41 of them had received training for more than a year. Concerning musical training, all groups were comparable, with statistically no significant differences between them.

Table S1: Music preference questionnaire descriptive statistics

Variables	Group A	Group B	Group C	Group D	*p*-Value
	N=37 (%)	N=36 (%)	N=36 (%)	N=35 (%)	
**Training in Music**					
**Yes**	17 (45.9)	14 (38.9)	11 (30.6)	12 (34.3)	0.562
**No**	20 (54.1)	22 (61.1)	25 (69.4)	23 (65.7)	
**The trained genre of music**					
**Indian**	14 (82.4)	11 (78.6)	9 (81.8)	10 (83.3)	0.898
**Western**	2 (11.8)	2 (14.3)	1 (9.1)	1 (8.3)	
**Both**	1 (5.9)	1 (7.1)	1 (9.1)	1 (8.3)	
**Duration of training in music**					
**≤1 year**	2 (11.8)	4 (28.6)	3 (27.3)	2 (16.7)	0.619
**> 1 year**	15 (88.2)	10 (71.4)	8 (72.7)	10 (83.3)	

Note:

a) N is the number of participants in each group.
b) All the values of the two groups are in absolute values and in parenthesis are in percentages.
c) *p*-value < 0.05 was considered significant
d) *p* calculated using Chi-square / Fisher exact test.

S3. Salivary Stress Markers

Table S2: Post hoc analysis of sAA and sCort

	Group			Mean Difference (I-J)	Std. Error	*p*-Value.[b]	95% Confidence Interval for Difference[b]	
							Lower Bound	Upper Bound
**Salivary Cortisol in U/mL**	D	Pre	Dur	.079*	.024	.007	.019	.139
			Post	-.021	.019	.807	-.069	.027
		Dur	Post	-.100*	.021	.000	-.155	-.046
**Salivary Alpha-Amylase in U/mL**	A	Pre	Dur	66.154*	6.542	.000	49.456	82.852
		Dur	Post	-80.228*	10.720	.000	-107.589	-52.866
	B	Pre	Dur	73.108*	7.078	.000	54.995	91.220
			Post	-26.663*	8.962	.019	-49.597	-3.729
		Dur	Post	-99.771*	10.417	.000	-126.427	-73.115
	C	Pre	Dur	63.538*	6.149	.000	47.541	79.534
		Dur	Post	-74.661*	7.942	.000	-95.322	-54.001
	D	Pre	Dur	60.913*	5.104	.000	47.851	73.974
		Dur	Post	-72.714*	7.504	.000	-91.917	-53.510
	Based on estimated marginal means							
	*. The mean difference is significant at the .05 level.							
	b. Adjustment for multiple comparisons: Bonferroni.							

S4. Blood pressure and Heart Rate

On comparison of pre-intervention levels of all SBP, DBP, and HR across the groups, it was observed that they were comparable (SBP, P = 0.708; DBP, P = 0.371; and HR, P = 0.741). Between the groups, we did not find any statistically significant difference (SBP: group A, P = 0.794; B, P = 0.416; C, P = 0.234; D, P = 0.215; DBP: group A, P = 0.208; B, P = 0.484; C, P = 0.622; D, P = 0.429). On RM-ANOVA analysis, no significant differences were observed in SBP in any of the groups (See Figure 4a,4b in main text). However, there was a continuous trend of reduction in SBP in groups C and D. In group B, SBP was reduced only during the intervention and returned to pre-intervention levels after the intervention. In group A, SBP increased slightly with music. Similar to SBP, the DBP change was statistically not significant in any of the groups (See Figure 4c,4d in the main text). A trend towards sustained rise was observed in groups B and C with and after the intervention, while in group A, DBP reduced with music and returned to baseline after the intervention. In the control group, DBP increased slightly with the intervention, followed by a mild reduction after the intervention. In groups, B, C, and D, a sustained drop in HR was observed throughout the 30-minute duration, with group C having a higher drop in HR during the intervention (See Figure 4e,4f in main text).

Table S3a: Comparison of systolic blood pressure (in mm Hg), diastolic blood pressure (in mm of Hg) and Heart rate between four intervention groups (pre, during and post music intervention)

					Quartiles			
	**Group**	Time	Mean	SD	25	50	75	*P*
Systolic Blood Pressure (in mm Hg)	**A (N=37)**	Pre	107.33	9.7	104	114	123	0.818
		Dur	107.81	9.2	106	113.5	120.8	
		Post	107.64	10.5	105	115	124.4	
	**B (N=36)**	Pre	107.22	9.0	100	107.5	112.75	0.416
		Dur	106.53	10.4	99	107	113.5	
		Post	107.44	10.0	100.25	106.5	111.75	
	**C (N=36)**	Pre	108.47	10.5	100.25	106	114	0.236
		Dur	106.94	10.4	100.25	105.5	112.5	
		Post	106.28	12.2	100.25	107	115	
	**D (N=35)**	Pre	105.71	10.0	99	107	113	0.22
		Dur	105.35	10.3	98	108	113	
		Post	103.76	11.4	95	104	113	
Diastolic Blood Pressure (in mm of Hg)	**A (N=37)**	Pre	73.75	7.1	71	79.5	85	0.163
		Dur	72.03	5.2	71	75.5	80	
		Post	72.47	7.3	72	76.5	82.8	
	**B (N=36)**	Pre	71.03	6.7	65.5	73.5	76	0.484
		Dur	71.44	7.3	69	72	76	
		Post	72.03	7.3	67.25	72	78.75	
	**C (N=36)**	Pre	71.08	6.7	65	71	75.75	0.622
		Dur	71.5	8.2	67	70.5	75.75	
		Post	72.11	8.5	66	74	77.75	
	**D (N=35)**	Pre	71.79	6.1	68	73	77	0.403
		Dur	73.03	6.8	68	74	77	
		Post	72.41	7.8	65	73	79	
Heart Rate (in bpm)	**A (N=37)**	Pre	75.75	11.5	76	83	90.2	**0.031**
		Dur	76.31	11.7	77	86	90.4	

		Post	73.28	12.6	75	80.5	88	
	B (N=36)	Pre	73.61	13.5	63.25	70.5	80.25	0.478
		Dur	73.28	15.1	60	71	83	
		Post	72.11	12.9	60.25	71	77.75	
	C (N=36)	Pre	73.67	14.7	66.25	71.5	80.75	0.279
		Dur	71.17	12.1	62.25	71	79.75	
		Post	70.92	12.4	63	69.5	80.75	
	D (N=35)	Pre	72.59	12.9	65	71	79	0.74
		Dur	72.18	8.8	68	73	76	
		Post	71.62	9.6	65	72	76	

Note:

a) N is the number of participants in each group.
b) All the values are in mean and standard deviation (SD)—univariate ANOVA.
c) p-value < 0.05 was considered significant— Levene's test of equality
d) p calculated using RM-ANOVA.

Table S3b: Pairwise comparison of HR in group A based on time (Pre =1 , Dur = 2, Post = 3)

RANDOM GROUP	(I) TIME	(J) TIME	Mean Difference (I-J)	Std. Error	p-value[a]	95% Confidence Interval for Difference[a]	
						Lower Bound	Upper Bound
A	1	2	-.676	1.084	1.000	-3.397	2.045
		3	2.676	1.403	.193	-.846	6.197
	**2**	1	.676	1.084	1.000	-2.045	3.397
		**3**	**3.351**	1.424	.073*	-.225	6.928
	3	1	-2.676	1.403	.193	-6.197	.846
		2	-3.351	1.424	.073	-6.928	.225
Based on estimated marginal means; 1 is pre-intervention, 2 is during the intervention, and 3 is post-intervention.							
a. Adjustment for multiple comparisons: Bonferroni.							

S5. Heart Rate Variability

Table S4: Comparison of time-domain parameters of HRV between the four groups

			**Mean**	**SD**	**Min**	**Max**	**Percentiles**			**$F(df_{time}, df_{error})$ = F value**	***p***
							**25**	**50**	**75**		
**Mean NN (ms)**	A	Pre	832.57	118.5	620.38	1086.49	743.07	821.55	895.36	(2,70)=8.854	**<0.0001**
		Dur	844.19	127.7	632.18	1121.28	758.83	830.08	905.14		
		Post	855.23	126.7	619.8	1125.15	776.01	839.84	922.31		
	B	Pre	844.49	143.8	557.96	1151.72	746.13	860.74	945.53	(1.585,53.874)=16.908	**<0.0001**
		Dur	869	149.3	573.24	1207.65	760.84	887.42	1002.58		
		Post	877.22	147.6	607.72	1202.98	768.23	877.55	1000.37		
	C	Pre	861.24	129.7	598.65	1179.46	782.3	837.65	953.48	(2,70)=3.642	**0.031**
		Dur	869.06	127.2	595.91	1196.59	776.75	859.66	935.82		
		Post	884.7	133.1	603.84	1233.71	783.54	865.35	951.85		
	D	Pre	843.94	131.3	626.76	1119.1	751.33	811.11	953.43	(1.247,41.143)=5.844	**0.015**
		Dur	862.68	131.2	628.03	1154.47	790.99	828.34	952.19		

		Post	863.42	128.4	587.25	1162.77	790.9	849.58	934.38		
Mean HR	A	Pre	73.44	10.1	55.22	96.71	67.01	73.03	80.75	(2,70)=5.256	**0.008**
		Dur	72.63	10.7	53.51	94.91	66.29	72.28	79.07		
		Post	71.65	10.5	53.33	96.81	65.06	71.45	77.33		
	B	Pre	73.24	13.5	52.1	107.54	63.46	69.72	80.42	(1.625,55.249)=14.987	**<0.0001**
		Dur	71.2	13.2	49.68	104.67	59.85	67.61	78.87		
		Post	70.41	12.5	49.88	98.73	59.98	68.37	78.1		
	C	Pre	71.2	10.7	50.87	100.23	62.94	71.63	76.7	(2,70)=3.907	**0.025**
		Dur	70.44	10.1	50.14	100.69	64.13	69.8	77.25		
		Post	69.28	10.2	48.63	99.36	63.05	69.34	76.58		
	D	Pre	72.78	11.3	53.61	95.73	63.21	74.16	79.97	(1.177,38.837)=5.109	**0.024**
		Dur	71.08	10.5	51.97	95.54	63.39	72.58	76.61		
		Post	70.99	10.6	51.6	102.17	65.4	70.68	75.88		
SDNN	A	Pre	66.07	30.5	19.76	152.49	45.92	61.3	78.39	(2,70)=11.208	**<0.001**
		Dur	64.38	32.1	21.94	167.05	44.35	57.71	69.56		
		Post	74.41	36.1	19.89	165.59	53.12	64.9	94.15		
	B	Pre	63.9	27.9	20.49	125.52	40.1	63.3	82.46	(2,68)=4.066	**0.022**
		Dur	67.11	31.9	16.26	148.53	39.36	66.13	91.04		
		Post	71.2	31.9	19.46	147.24	40.29	72.8	94.73		
	C	Pre	71.83	34.7	28.58	173.05	48.66	64.57	87.48	(2,70)=9.068	**<0.001**
		Dur	66.59	29.9	25.19	136.92	42.75	63.22	79.99		
		Post	78.82	38.5	30.6	182.07	53	68.44	96.62		
	D	Pre	62.12	24.4	20.56	129.43	41.66	58.32	80.07	(2,66)=8.580	**<0.001**
		Dur	64.97	24.1	25.33	128.67	43.92	65.06	83.2		
		Post	71.63	27.2	22.26	132.79	49.61	67.44	90.62		
RMSSD	A	Pre	65.2	45.8	14.76	230.66	37.74	51.57	75.6	(2,70)=4.619	**0.013**
		Dur	63.67	50.3	10	260.82	32.57	50.06	77.09		
		Post	73.15	55.2	13.58	258.84	34.08	56.69	84.53		
	B	Pre	61.71	41.7	8.91	167.28	28.73	58.43	81.11	(2,68)=3.207	0.05
		Dur	66.74	48.3	7.47	191.13	29.97	64.91	80.94		
		Post	67.27	45.3	10.57	195.64	29.56	64.39	87.41		
	C	Pre	70.39	48.3	13.14	185.39	34.76	53.4	97.38	(2,70)=3.34	**0.041**
		Dur	67.64	47.7	14.79	216.71	34.63	54.36	83.76		
		Post	77.34	55.7	17.36	277.13	38.03	55.32	97.57		
	D	Pre	58.99	32.0	11.4	127.27	35.37	48.15	83.45	(2,66)=6.31	**0.003**
		Dur	62.6	31.5	12.18	131.82	37.51	59.58	83.91		
		Post	66.96	33.7	11.4	147.01	40.22	62.03	95.94		
NN50	A	Pre	226.19	132.6	1	486	110.75	253.5	310.75	(2,70)=1.363	0.263
		Dur	213	140.1	0	460	71	213	302.5		
		Post	231.56	144.2	1	484	85.5	231.5	347		
	B	Pre	223.31	185.6	0	566	52	223.5	391.5	(1.435,48.794)=0.248	0.707
		Dur	215	162.9	0	528	68.5	213.5	330		
		Post	219.94	160.1	1	530	74	215	312		
	C	Pre	258.42	190.6	2	664	92.25	198.5	425.5	(1.66,58.243)=0.034	0.946
		Dur	257.28	171.7	3	536	94	270.5	436.25		
		Post	254.64	142.4	12	544	125.5	243	376.25		
	D	Pre	236.47	183.1	1	890	98	201.5	356.5	(1.178,58.352)=0.204	0.789
		Dur	246.94	162.0	2	746	118.5	255	341		
		Post	247.15	137.2	0	496	135.75	280	344		
[illegible]	A	Pre	30.45	19.9	0.12	78.41	13.46	30.6	42.34	(2,70)=1.588	0.212

	Dur	30.86	22.4	0	82.64	9.31	31.47	44.11		
	Post	32.97	22.5	0.1	80.66	10.86	30.63	41.06		
B	Pre	32.19	26.2	0	83.07	6.84	34.25	52.07	(1.393,47.375)=2.147	0.142
	Dur	33.34	26.5	0	80.4	7.33	35.1	52.76		
	Post	35.04	26.1	0.1	82.66	8.44	37.5	55.97		
C	Pre	34.99	24.7	0.21	75.43	10.87	33.18	54.16	(1.756,61.469)=3.128	0.06
	Dur	35.13	24.9	0.31	82.59	12.33	33.62	52.07		
	Post	37.92	23.4	1.15	79.07	16.76	37.53	56.02		
D	Pre	31.61	22.5	0.1	71.77	12.06	26.27	52.85	(1.235,40.765)=3.088	0.078
	Dur	33.54	21.9	0.19	72.01	14.58	34.54	52.39		
	Post	35.34	21.1	0	71.62	16.78	35.3	52.54		

Table S5: Comparison of frequency-domain parameters of HRV between the four groups

			Mean	SD	Min	Max	Percentiles			$F(df_{time}, df_{error})$ = F value	*p*
							25	50	75		
TP (ms2)	A	Pre	5095.95	5419.2	420.16	22146.9	2011.06	3380.3	5599.52	(2,70)=7.291	**0.001**
		Dur	4913.12	5759.5	427.13	27246.6	1433.55	3048.35	4568.31		
		Post	6510.2	6838.6	335.16	26477.4	2637.89	3680.8	8644.46		
	B	Pre	4495.09	3770.9	338.84	14823.3	1449.76	3737.17	6117.14	(1.748,61.167)=4.421	**0.020**
		Dur	5048.44	4646.1	258.78	19947.9	1326.04	3668.72	7551.1		
		Post	5875	5286.2	303.78	22966.3	1488.67	4798.3	7988.89		
	C	Pre	6149.24	6509.2	539.63	30386.7	2190.71	4216.17	7983.97	(2,70)=4.702	**0.012**
		Dur	5291.66	5276.2	546.98	20559.6	1510.47	3755.9	6523.42		
		Post	7502.7	8286.2	815.09	33352.4	2270.37	4306.67	9433.66		
	D	Pre	4177.44	3342.3	395.44	14789.2	1641.55	3070.9	6440.53	(2,66)=3.8	**0.027**
		Dur	4567.27	3465.4	645.93	15867.7	1993.28	3449.98	6487.22		
		Post	5255.51	3774.2	432.59	17376	2409.59	4113.7	7291.77		
VLF (ms2)	A	Pre	1060.76	641.9	194.13	2695.84	644.46	902.33	1479.56	(1.595,55.824)=3.127	0.063
		Dur	1117.27	1101.3	157.76	5277.33	448.31	776.43	1164.49		
		Post	1537.73	1568.3	98.11	8131.27	599.09	1127.61	1575.58		
	B	Pre	1113.18	935.5	146.12	3957.64	402.58	746.13	1552.25	(2,70)=4.595	**0.013**
		Dur	1300.72	1047.0	119.82	3847.22	547.56	866.94	1876.43		
		Post	1744.05	1689.5	69.21	7374.79	574.94	1321.68	1798.24		
	C	Pre	1878.68	2249.1	196.83	11816.5	641.15	1129.85	2063.46	(2,70)=5.313	**0.007**
		Dur	1418.72	1908.4	93.97	11297.7	464.35	886.17	1785.35		
		Post	2071.34	2887.3	142.9	15538	625.55	1335.72	2295.61		
	D	Pre	1151.48	1038.4	77.69	5336.71	489.1	892.08	1402.54	(2,66)=0.806	0.451
		Dur	1336.48	1264.7	320.42	7449.73	633.41	1068.22	1632.61		
		Post	1404.73	1012.3	100.14	4537.77	736.91	1225.15	1683.94		
LF (ms2)	A	Pre	1364.42	1474.0	103.11	7839.03	388.75	924.06	1784.1	(1.330,46.538)=4.71	**0.026**
		Dur	1220.28	1409.4	142.11	7303.4	321.76	851.67	1464.24		
		Post	1771.5	1830.6	134.03	7245.26	524.82	1140.08	2012.94		
	B	Pre	1131.91	1010.8	61.49	4601.4	427.35	857.15	1556.1	(1.439,50.378)=2.502	0.108
		Dur	1214.31	1135.3	81.89	4121.94	381.04	709.15	1927.15		
		Post	1524.86	1883.8	167.12	10786.4	571.66	1047.09	1897.06		
	C	Pre	1503.04	1628.1	159.18	7876.05	585	907.87	1820.27	(1.284,44.942)=4.559	**0.029**
		Dur	1232.27	1063.5	111.19	4491.08	470.35	980.56	1726.92		
		Post	1960.42	2150.8	257.78	11504	642.69	1050	2973.81		

	D	Pre	1171.37	1188.6	93.36	6494.83	420.6	877.64	1518.69	(1.555,53.393)=1.87	0.172
		Dur	1295.99	1266.7	168.93	5964.73	486.54	881.23	1587.72		
		Post	1539.95	1426.8	217.33	7140.09	663.82	1156.17	2002.34		
**LF (nu)**	A	Pre	40.44	15.8	10.04	79.63	29.14	41.43	51.12	(2,70)=4.535	**0.014**
		Dur	39.56	16.3	10.1	74.73	29.34	42.78	50.25		
		Post	43.83	19.3	6.18	82.62	25.72	46.46	58.05		
	B	Pre	43.25	21.1	9.16	85.14	25.55	39.75	57.9	(2,70)=0.746	0.478
		Dur	42.41	21.7	9.94	84.62	25.86	38.38	58.96		
		Post	44.33	20.7	13.5	79.48	25.8	40.67	62.77		
	C	Pre	42.29	17.4	14.6	83	29.69	42.93	52.42	(2,70)=2.141	0.125
		Dur	40.66	18.8	11.87	87.23	25.97	37.84	49.43		
		Post	43.48	17.8	6.55	81.41	27.44	45.74	55.97		
	D	Pre	41.73	13.9	20.24	67.72	30.71	41.35	52.37	(2,66)=0.87	0.424
		Dur	41.4	15.4	20.31	79.86	28.86	39.13	51.02		
		Post	43.48	15.1	17.8	70.97	31.55	41.91	52.16		
**HF (ms2)**	A	Pre	2300.53	3508.0	102.7	15196.9	407.58	1223.75	2255.82	(2,70)=2.106	0.129
		Dur	2205.29	3845.6	98.3	20973.8	411.45	977.11	1922.42		
		Post	2724	4135.8	91.38	19746.4	492.49	1151.97	2155.6		
	B	Pre	1952.82	2494.1	33.24	11426.6	394.03	1232.09	3079.13	(2,70)=1.677	0.194
		Dur	2223.82	3020.5	22.34	14610.3	323.41	1621.43	2601.11		
		Post	2312.33	2959.0	47.96	13970	399.83	1592.69	2725.47		
	C	Pre	2069.48	2154.3	49.18	8027.73	502.94	1177.53	3043.25	(2,70)=2.227	0.115
		Dur	2150.6	2591.3	97.16	10952.5	476.94	1084.4	2833.33		
		Post	2645.22	2961.8	158.65	11548.2	534.86	1313.53	4035.13		
	D	Pre	1591.07	1545.0	75.73	5533.11	398.95	1067.53	2321.29	(1.190,39.274)=4.203	**0.041**
		Dur	1709.74	1479.9	87.44	5035.98	567.68	1302.84	2704.98		
		Post	2047.83	1897.6	97.09	7072.91	558.5	1513.38	3154.41		
**HF (nu)**	A	Pre	49.14	14.6	14.43	76.68	40.59	49.39	59.21	(2,70)=2.402	0.098
		Dur	49.43	15.4	22.45	78.69	40.78	49.36	59.43		
		Post	46.47	17.1	12.87	78.73	35.35	41.79	57.72		
	B	Pre	48.8	19.1	11.92	82.58	34.39	51.84	59.4	(2,70)=0.401	0.672
		Dur	50.02	19.1	12.05	78.64	34.13	54.58	63.74		
		Post	49.19	18.8	16.65	80.95	32.09	53.98	64.43		
	C	Pre	50.02	16.2	15.33	80.01	40.03	49.87	62.66	(2,70)=1.759	0.18
		Dur	52.01	18.0	11.5	81.19	43.68	53.86	65.6		
		Post	49.38	17.4	16.37	81.26	38.23	47.1	65.53		
	D	Pre	49.34	14.3	17.34	72.88	35.2	51.35	59.64	(1.716,56.627)=0.775	0.448
		Dur	51.37	14.8	19.02	71.32	38.57	52.97	64.92		
		Post	49.44	14.0	25.14	74.29	36.69	52.51	60.39		
**LF/HF**	A	Pre	1.01	0.8	0.15	4.16	0.57	0.83	1.26	(1.457,51.004)=4.385	**0.028**
		Dur	0.97	0.7	0.14	3.24	0.57	0.89	1.11		
		Post	1.23	1.0	0.08	5.25	0.61	1.09	1.5		
	B	Pre	1.39	1.6	0.12	7.14	0.41	0.76	1.71	(1.474,51.579)=0.039	0.922
		Dur	1.38	1.6	0.13	7.02	0.41	0.7	1.77		
		Post	1.35	1.3	0.17	4.77	0.4	0.73	2.05		
	C	Pre	1.14	1.2	0.19	5.42	0.47	0.86	1.27	(1.672,55.165)=0.043	0.936
		Dur	1.18	1.5	0.18	7.59	0.44	0.67	1.1		
		Post	1.16	1.1	0.22	4.97	0.42	0.95	1.44		
	D	Pre	1.03	0.7	0.28	3.45	0.5	0.82	1.43	(1.676,58.663)=0.242	0.746
		Dur	1.02	0.8	0.3	4.2	0.44	0.75	1.27		

Post	1.09	0.8	0.24	2.99	0.52	0.79	1.42

Table S6: ANCOVA analysis of VLF ms² based on drinking history

	Alcoholism history	F	*p*-value time	F	*p*-value Time*group
**VLF (ms²)**	Yes	0.833	0.445	2.828	0.027
	No	11.121	<0.0001	0.836	0.534
**Log VLF (ms²)**	Yes	0.200	0.820	1.438	0.233
	No	11.315	<0.0001	1.893	0.083

Supplementary File References

[1] D. Raja, 'Deepak Raja's world of Hindustani Music: Raga Miya-ki Todi.... reluctant differentiation', *Deepak Raja's world of Hindustani Music*, Apr. 24, 2011. http://swaratala.blogspot.com/2011/04/raga-miya-ki-todi-reluctant.html (accessed Mar. 19, 2020).

[2] 'Film Songs in Rag Mian Ki Todi'. https://chandrakantha.com/raga_raag/film_song_raga/mian_ki_todi.shtml (accessed Mar. 19, 2020).

[3] 'The Raga Guide - Malkauns', Jun. 20, 2009. https://web.archive.org/web/20090620030217/http://www.wyastone.co.uk/nrl/world/raga/malkauns.html (accessed Mar. 19, 2020).

[4] 'Raag Puriya - Indian Classical Music - Tanarang.com'. http://www.tanarang.com/english/puriya_eng.htm (accessed Mar. 19, 2020).

[5] 'Puriya', *Wikipedia*. Nov. 20, 2019. Accessed: Mar. 19, 2020. [Online]. Available: https://en.wikipedia.org/w/index.php?title=Puriya&oldid=927072231
